# Targeting SCAMP2 by a natural product auxarconjugatin B for glioblastoma therapy *via* restoring aspartate metabolic flux

**DOI:** 10.1016/j.apsb.2026.02.018

**Published:** 2026-02-27

**Authors:** Changhui Shang, Wan Li, Qianlun Pu, Bingyu Liu, Chen Zhang, Yiqing Tan, Yihui Yang, Dan Du, Jinhua Wang, Youcai Hu

**Affiliations:** a State Key Laboratory of Bioactive Substance and Function of Natural Medicines, Institute of Materia Medica, Chinese Academy of Medical Sciences & Peking Union Medical College, Beijing 100050, China; b Advanced Mass Spectrometry Center, Research Core Facility, Frontiers Science Center for Disease-related Molecular Network, West China Hospital, Sichuan University, Chengdu 610041, China

**Keywords:** Auxarconjugatin B, Glioblastoma, SCAMP2, ABPP, Aspartate, Metabolic flux, Aspartate transporters, SLC1A3

## Abstract

Glioblastoma (GBM), the most aggressive primary brain malignancy, presents an urgent need for novel therapeutic targets addressing metabolic reprogramming in tumor progression. Natural product-based molecular probes have emerged as powerful tools for target discovery and mechanistic elucidation in cancer biology. Here, we identified the fungal polyketide auxarconjugatin B (AUX-B) as a potent inhibitor of GBM proliferation through both *in vitro* and *in vivo* models. Chemical biology strategies revealed secretory carrier membrane protein 2 (SCAMP2) as the covalent cellular target of AUX-B. SCAMP2 exhibited significant overexpression in human GBM specimens and orthotopic GBM mouse models, correlating with tumor progression. Mechanistic investigations demonstrated that SCAMP2 orchestrates metabolic reprogramming through the regulation of aspartate transporters (solute carrier family 1 member 3 and solute carrier family 25 member 12) and asparagine synthetase, thereby sustaining aspartate metabolic flux critical for GBM growth. AUX-B-mediated reduction of SCAMP2 effectively disrupted this pathogenic metabolic network, leading to a decrease in intracellular aspartate levels. Our findings establish SCAMP2 as a novel therapeutic target in GBM and characterize AUX-B as a new SCAMP2 inhibitor with translational potential through metabolic modulation.

## Graphical abstract

Auxarconjugatin B was an anti-glioblastoma candidate targeting SCAMP2, which was further identified as a novel therapeutic target primarily through regulation of aspartate transport, rewriting aspartate metabolism in glioblastoma.

**Figure:**
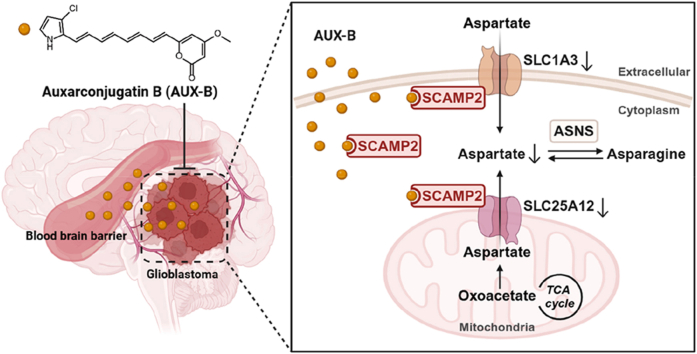

## 1 Introduction

Glioma is a primary central nervous system tumor originating from glial cells, which constitutes nearly 30% of all primary brain tumors and 80% of malignant brain tumors, accounting for most deaths associated with primary brain tumors^1^. The World Health Organization classifies central nervous system gliomas into grades I–IV, with glioblastoma (GBM) being the most aggressive and lethal grade IV tumor, characterized by rapid growth, high invasiveness, and a poor prognosis^2^. The current standard treatment regimen involves maximal safe surgical resection followed by radiotherapy and adjuvant temozolomide chemotherapy^3^. Despite this multimodal approach, significant challenges remain in GBM treatment, including incomplete surgical resection, therapeutic drug resistance, the blood–brain barrier, immunosuppressive microenvironment, and tumor heterogeneity^4^. Notably, the tumor metabolic reprogramming not only exerts a remarkable influence on tumor progression but also has an impact on therapeutic drug resistance and the immune microenvironment^5^. Thus, targeting metabolic reprogramming and potential targets might be a crucial means to enhance the clinical outcomes of GBM patients.

Metabolic reprogramming, a hallmark of cancer, has emerged as a critical area of research in GBM biology^6^. Profound metabolic alterations, such as enhanced glycolysis and pentose phosphate pathway, adaptively intensified lipid and amino acid metabolism, are contributing to the immunosuppressive microenvironment, therapeutic resistance, and tumor progression^7^. Aspartate, an acidic non-essential amino acid, can be synthesized by glutamic-oxaloacetic transaminases 2 and/or imported from external sources *via* the plasma membrane glutamate and aspartate transporter solute carrier family 1 member 3 (SLC1A3)^8^^,^^9^. Cytosolic aspartate is a precursor for purines, pyrimidines, and asparagine biosynthesis, and is also involved in the urea cycle and malate-aspartate shuttle through solute carrier family 25 member 12 (SLC25A12) or solute carrier family 25 member 13, an aspartate-glutamate carrier^10^, ^11^, ^12^. Although glutamate metabolism and trafficking are profoundly altered in gliomas^13^^,^^14^, the utilization of aspartate by cells remains poorly understood. Secretory carrier membrane protein 2 (SCAMP2) is ubiquitously expressed in eukaryotic cells and plays critical roles in intracellular membrane trafficking and secretory processes^15^^,^^16^. Dysregulation of SCAMP2 may contribute to acute myeloid leukemia, diabetes, and neurological disorders^17^, ^18^, ^19^. Although previous studies have demonstrated functional interactions between SCAMP2 and the dopamine transporter^20^, its role in GBM and its regulation of aspartate transport remain unclear.

Natural products have long been a cornerstone of drug discovery and chemical biology research due to their structural diversity and potent biological activities^21^^,^^22^. Many natural products have served as “molecular probes” to uncover novel drug targets, providing insights into disease mechanisms and biological processes^23^. For instance, compounds like rapamycin and paclitaxel have not only become essential drugs but have also revealed key targets such as mTOR and tubulin, respectively^24^^,^^25^. Auxarconjugatin B (AUX-B), a polyenylpyrrole-type polyketide isolated from fungi of *Auxarthron* species^26^^,^^27^. and its serial analogs have been reported to exhibit remarkable antitumor activities against leukemia, melanoma, ovarian cancer, and lung cancer^28^, ^29^, ^30^. However, the role and target of AUX-B in GBM remain unclear.

Here, we aim to explore the role of AUX-B in GBM treatment and its potential targets for further drug discovery. Firstly, the inhibitory effects of AUX-B on the proliferation of GBM *in vitro* and *in vivo* were evaluated on two cell lines, U87 and U251, as well as two xenograft tumor models. Secondly, we designed a probe for AUX-B and employed click chemistry to identify its covalent target, SCAMP2. The protective effects of the *SCAMP2* gene on GBM proliferation were subsequently elucidated. Mass imaging and metabolic flux analysis were then conducted to investigate the metabolic reprogramming regulated by AUX-B and SCAMP2, revealing abnormalities in aspartate abundance and transport associated with asparagine synthetase (ASNS), as well as the aspartate transporters SLC1A3 and SLC25A12. Lastly, co-immunoprecipitation assays and fluorescence colocalization experiments confirmed that SCAMP2 mediated by AUX-B interacted with SLC1A3 and SLC25A12. Altogether, the natural product AUX-B exerts therapeutic effects on GBM by targeting SCAMP2 and majorly disrupting its interaction with aspartate transporters, thereby depleting intracellular aspartate levels.

## 2 Materials and methods

### 2.1 Cell culture and cell biology experiment

The human GBM cell lines U87-MG were purchased form Wuhan Pricella Biotechnology Co., Ltd. (Wuhan, China), and U251-MG cells were kindly provided by the Cancer Hospital of the Chinese Academy of Medical Sciences, HEK-293T cells were sourced from the American Type Culture Collection (ATCC, Manassas, VA, USA), theses three types of cells were cultured in Dulbecco’s modified Eagle’s medium (DMEM) with 10% FBS (164210-50, Procell, Wuhan, China). The murine glioma cell lines GL261 were obtained from BeNa Culture Collection (Shangcheng, Henan, China) and cultured in DMEM with 10% FBS, 1% glutamine (200 mmol/L), and 1% HEPES (1 mol/L). All cells were cultured in a humidified 5% CO_2_ incubator at 37 °C.

#### 2.1.1 Cell viability assay

Cells were seeded at a density of 3000 cells per well in a 96-well plate. The following day, AUX-B was added to reach final concentrations of 0, 0.01, 0.03, 0.1, 0.3, 1, 3, 10, and 30 μmol/L, respectively. After 24, 48, and 72 h, cell viability was evaluated using the CCK-8 kit (C0040, Beyotime, Shanghai, China), and the IC_50_ values were calculated using GraphPad software.

#### 2.1.2 Cell cycle assay

Cells were collected, fixed, and then resuspended in a propidium iodide solution (C0080, Solarbio, Beijing, China). The cell cycles were analyzed using a BD C6 Flow Cytometer (BD, San Diego, CA, USA). The distribution of the cell cycle was analyzed by Flow Jo_V10 software.

#### 2.1.3 EdU DNA synthesis assay

Cells were seeded at a density of 6000 cells per well in a 96-well plate. The next day, AUX-B was added with a final concentration of 0, 0.25, 0.5, and 1 μmol/L, respectively. After 24 or 48 h, the DNA synthesis rate of GBM cells was evaluated using the Cell-Light EdU Apollo567 In Vitro Kit (C10310-1, Ribobio, Guanzhou, China) according to the manufacturer’s instructions, and images were captured using a Nikon microscope.

#### 2.1.4 Transwell assay

For the migration assay, cells were harvested and suspended in DMEM complete medium at a density of 5 × 10^4^/mL. A volume of 300 μL of this cell suspension was added to the upper chamber of a transwell plate (33342, Corning Costar, Kennebunk, USA), while 1 mL of DMEM complete medium was added to the lower chamber. After 3 h, the upper culture medium was replaced with FBS-free DMEM containing AUX-B at concentrations of 0, 0.25, 0.5, and 1 μmol/L, respectively. Following a 21-h incubation, the cells that migrated through the polycarbonate membrane were fixed with 4% paraformaldehyde and stained with 1% crystal violet (G1062, Solarbio, Beijing, China).

For the invasion assay, Matrigel (354234, Corning Costar, Kennebunk, USA) was diluted in a ratio of 1:7 using FBS-free DMEM and used to cover the upper surface of the membrane, and the remaining procedures were the same as those used in the migration assay.

#### 2.1.5 Plate cloning experiment

Cells were plated in a 6-well plate at a density of 500 cells per well. The following day, the medium was replaced with fresh complete DMEM containing AUX-B at concentrations of 0, 0.25, 0.5, and 1 μmol/L, respectively. After incubating for 2–3 weeks, the cells were fixed using 4% paraformaldehyde and stained with 1% crystal violet (G1062, Solarbio, Beijing, China).

#### 2.1.6 Cell transfection assay

For cell transfection, cells were seeded and transfected with the plasmid by using Lipofectamine 3000 reagent (L3000015, Invitrogen, CA, USA). After being transfected for 24 h, treated with AUX-B or not, samples were observed for their localization situation by laser confocal microscope, or collected for co-immunoprecipitation. All plasmids were purchased from Youbio Biological Technology Co., Ltd., Changsha, China.

#### 2.1.7 Cell infection assay

For cell transduction, SCAMP2 knockout and SCAMP2-overexpressing in U87 were generated by lentiviral transduction and selected by 1 μg/mL puromycin. All sg-SCAMP2 sequences are listed as follows: sg-SCAMP2#2: 5′-aattccgccaggccgccctg-3′, sg-SCAMP2#3: 5′-actgatggctggagaaccgc-3′. Lentiviruses were purchased from Genechem Co., Ltd., Shanghai, China.

#### 2.1.8 RNA extraction and RT-qPCR

Total RNA was extracted using the Total RNA Extraction Kit (R0026, Beyotime, Shanghai, China). Complementary DNA (cDNA) was reverse-transcribed from total RNA by using Super RT cDNA Synthesis Kit (CW0741M, CWBIO, Beijing, China). The qPCR analysis was conducted by RNase-free water (R1600, Solarbio, Beijing, China), Magic SYBR Mixture (CW3008M, CWBIO, Beijing, China), and primers (purchased from Beijing Tsingke Biology Co., Ltd.) on a CFX thermocycler (Bio-Rad, Hercules, CA, USA). The primer sequences are listed in Supporting Information Table S1.

#### 2.1.9 Western blot

Cells were harvested and lysed in RIPA lysis buffer (C1053-100, Applygen, Beijing, China) containing protease inhibitors and phosphatase inhibitors. After centrifugation, the supernatant was collected, and the protein concentration was determined by the BCA protein assay kit (P0011, Beyotime, Shanghai, China). The supernatant was denatured by the SDS-PAGE Loading Buffer (P0285, Beyotime, Shanghai, China), separated by SDS-PAGE electrophoresis, and transferred to the PVDF membranes. Then the membranes were blocked by 5% fat-free milk, incubated with diluted primary antibodies at 4 °C overnight, and incubated with secondary antibodies at room temperature for 1 h. Finally, bands were visualized by Super ECL Detection Reagent (36208ES60, Yeasen, Shanghai, China). The primary antibodies and secondary antibodies information are listed in Supporting Information Table S2.

#### 2.1.10 Immunofluorescence

The cells were treated with 4% paraformaldehyde for fixation, followed by permeabilization using 0.1% Triton X-100 (20107ES76, Yeasen, Shanghai, China) and blocking with 3% BSA in PBS. Subsequently, the cells were incubated with primary antibodies at the suggested dilutions and then labeled with secondary antibodies at room temperature for 1 h. The nuclei were stained with Hoechst 33342 for 30 min (C11028, Beyotime, Shanghai, China). Finally, the cells were examined using a laser confocal microscope (Leica TCS SP8X, Leica, Germany).

#### 2.1.11 Co-immunoprecipitation (Co-IP)

The treated cells were lysed using IP lysis buffer (C1054, Applygen, Beijing, China) and then centrifuged. The supernatant was collected, and a portion was denatured with SDS-PAGE Loading Buffer and used as input for further analysis. For Co-IP, the supernatant was gently agitated overnight at 4 °C with magnetic beads specific to His (P2135, Beyotime, Shanghai, China), Myc (B26301, Selleckchem, Houston, TX, USA, and Flag (B26101, Selleckchem, Houston, TX, USA) tags. The next day, the beads were washed five times with TBST buffer and then mixed with SDS-PAGE Loading Buffer at 70 °C for 10 min. Finally, the input and Co-IP samples were analyzed using a Western blot.

#### 2.1.12 Microscale thermophoresis (MST) binding assay

The affinity of AUX-B with wild-type SCAMP2 and K130A mutant protein was separately determined *via* a commercial MST technology service offered by Zoonbio Biotechnology Co., Ltd. (Nanjing, China). The concise experimental procedures are as follows: The serially diluted ligand molecules (AUX-B, a volume of 10 μL) were individually combined with a constant-concentration (100 nmol/L) of fluorescently labeled wild-type SCAMP2 and K130A mutant, and incubated at room temperature for 5 min. Subsequently, the samples were loaded into a glass capillary tube and analyzed by the Monolith NT.115Pico instrument (Nano Temper Technologies GmbH, Munich, Germany) at 30 °C, with an excitation power of 80% and a medium LED power. The dissociation constant (*K*_D_) was computed and fitted using Nano Temper Analysis software. Each experimental group was repeated 3 times independently.

#### 2.1.13 Molecular dynamics (MD) simulation

Molecular dynamics calculations were performed *via* a commercial MST technology service offered by Phadcalc (www.phadcalc.com, developed by Chengdu Tianji Computing Technology Co., Ltd.) by using the academic version of Desmond 2021.1. First, the 3D structure of the SCAMP2 protein was predicted by AlphaFold 2, amino acids 1–71 of SCAMP2 were truncated, as this domain is a loop chain with low structural quality, and will interfere with the molecular dynamic simulation. Two systems were constructed: a wild-type system and another with manually added small-molecule modifications. All these processes were handled using Schrödinger. The OPLS4 force field was selected, with the phospholipid bilayer set as POPC and the TIP3P water model adopted. The protein and small molecules were placed in a cubic water box. In the simulation, the cutoffs for electrostatic interactions and van der Waals interactions were both set to 1.0 Å, and the time step was 2 fs. The temperature was maintained at 27 °C (300 K) and the pressure at 1.01325 bar. The established systems were first subjected to 100 ps of Brownian Dynamics under the NVT ensemble at 10 K. Subsequently, 12 ps of equilibrium dynamics under the NVT ensemble and 12 ps under the NPT ensemble were performed sequentially at 10 K, followed by 12 ps of NPT simulation with heavy-atom restraints and 24 ps of unrestrained NPT simulation. Finally, 200 ns of molecular dynamics sampling was conducted. Post-simulation analyses included calculations of RMSD, RMSF, SASA, and gyration radius.

### 2.2 Animal experiment

All animal experiments were performed in compliance with the Guide for the Care and Use of Laboratory Animals (NIH) and received approval from the Animal Ethics Committee of Peking Union Medical College and the Chinese Academy of Medical Sciences (No: 00004116, Beijing, China). Mice were housed in cages with a 12-h light cycle at a temperature of 23 ± 2 °C, with free access to food and water.

#### 2.2.1 Serum drug concentration analysis

The *in vivo* drug concentrations of AUX-B in blood and brain were primarily determined in C57 mice. Briefly, the C57 mice received an IP injection of a 15 mg/kg dose of AUX-B, which was dissolved in a solvent consisting of 5% DMSO, 5% HS15, and 90% normal saline. At 5, 15, 30 min, 1, 1.5, 2, and 4 h following administration, the serum samples were collected into tubes, and the entire brain was removed and used for AUX-B mass spectrometry imaging. After 30 min of standing at room temperature, the blood was centrifuged for 15 min at 1500×*g*, and the top serum was extracted. 50 μL serum was mixed with 200 μL acetonitrile on ice and vortexed thoroughly. The mixture was then kept at −20 °C for 30 min, ultrasonically cooled for 15 min, and then centrifuged for 20 min at 9400 × *g* and 4 °C. The supernatant was transferred to LC–MS tubes for analysis. Frozen brain tissue (∼50 mg) was homogenized with 1 mL of prechilled acetonitrile/MilliQ water (4:1) at 4 °C. Samples were vortexed, stood, sonicated, and centrifuged before they were analyzed by LC–MS. The concentration of AUX-B in the samples was calculated based on a standard curve with a series of standard AUX-B, which was added to blank serum and brain tissue homogenate and pre-treated with the samples to remove the impact of the matrix effect. The concentration of AUX-B in brain tissue was corrected by weight and protein concentration.

Tissue metabolites were imaged using the AFADESI-MSI platform equipped with a high-resolution quadrupole-orbitrap tandem mass spectrometer (Q-Exactive Plus; Thermo Fisher Scientific, Waltham, MA, USA). The fresh brain tissues were flash-frozen in liquid nitrogen for 10 s, laid flat in a cryo box, and stored at −80 °C until sectioned at 10 μm thickness using a cryostat microtome (Leica Microsystems, Wetzlar, Germany’s CM 1950). The tissue sections were mounted on the microscope slide and stored in closed containers at −80 °C until detection. An adjacent serial tissue section was used for HE staining analysis. Before the AFADESI-MSI analysis, the microscope slides were dried in a vacuum for 30 min. The experiment was conducted in positive and negative full mass modes at *m/z* 70–1000 with a resolution of 70,000. The spray solvent was used as a mixture of acetonitrile and water (8:2, *v*/*v*), with a 5 μL/min flow rate. The sprayer voltages were set at 7000 V/–4500 V, the extracting gas was set at 45 L/min, and the spraying gas (nitrogen) was set at 0.7 MPa. The tissue surface was continually scanned by a 3D electrical moving stage at a speed of 0.1 mm/s in the *x*-direction and a 0.15 mm vertical step in the *y*-direction. MassImager software was used for background subtraction and image reconstruction.

#### 2.2.2 *In vivo* pharmacodynamic evaluation of AUX-B

##### 2.2.2.1 Subcutaneous xenograft tumor model

In this study, 8-week-old NCG female mice (GemPharmatech, Jiangsu, China) were used. U87 cells were harvested, and 7 × 10^6^ cells were subcutaneously implanted into the right flank of the mice. Treatment commenced when the tumor volume reached 100 mm^3^. The tumor volume (*V*, in mm^3^) was determined using Eq. (1):

(1) *V* = 0.5 × *L* × *w*^2^

where *L* represents length and *w* represents width in mm. Following euthanasia, tumor tissues were gathered, weighed, and photographed.

Drug treatment: AUX-B was administered at doses of 1.5 mg/kg and 3.0 mg/kg twice daily for 24 consecutive days *via* intraperitoneal injection (bid, i.p.). 1% DMSO +1% Solution HS15 + 98% normal saline (NS) was used as the solvent.

##### 2.2.2.2 *In situ* xenograft tumor model

17–19 g BALB/c nude female mice (Charles River, Beijing, China) were employed in this study. The mice were intraperitoneally injected with 50 mg/kg sodium pentobarbital and fixed in the prone position with a stereoscope. The striatum was located using a stereotaxic apparatus (AP: +0.50 mm; ML: −2.0 mm; DV: −3.3 mm) and punctured with a cranial drill. Subsequently, 5 μL (5 × 10^5^ cells) of U87-MG cells were injected at a rate of 1 μL/min, with a 5-min wait to prevent any leakage of the tumor cell suspension. Treatment began on the fifth day. At the end of the experiments, the sagittal and coronal planes of the xenograft mice were imaged using a magnetic resonance scanner (Gto 3.01, Bulker). The volume of the intracranial tumor was calculated by summing the areas of various layers multiplied by the layer distance.

Drug treatment: AUX-B was administered at doses of 1.5 mg/kg and 3.0 mg/kg twice daily for 21 consecutive days *via* intraperitoneal injection (bid, i.p.). 1% DMSO +1% Solution HS15 + 98% NS was used as the solvent.

#### 2.2.3 In situ allograft tumor model

In this study, 8-week-old female C57BL/6J mice (from GemPharmatech, Jiangsu, China) were used. A total of 5 × 10^4^ GL261 cells in 5 μL were injected, and the subsequent procedures followed the same protocol as outlined in Section 5.2.2.2 for the *in situ* xenograft tumor model. After three weeks of transplantation, the mice were euthanized, and their brains were extracted and fixed in 4% paraformaldehyde for 24 h for further IHC experiments.

#### 2.2.4 *In vivo* evaluation of the tumorigenic ability of SCAMP2

In this study, female BALB/c nude mice weighing between 17 and 19 g (Charles River, Beijing, China) were used. U87-sgNC, U87-sgSCAMP2#2, and U87-sgSCAMP2#3 cells, each at a total of 1 × 10^7^ cells, were implanted subcutaneously into the right flank of the mice. Measurements of tumor volume and mouse weight commenced once the tumor volume reached 100 mm^3^ and were conducted twice a week until the average tumor volume grew to approximately 1000 mm^3^.

#### 2.2.5 H&E staining

Tissue sections embedded in paraffin were first deparaffinized using xylene (twice) and then rehydrated with a series of ethanol solutions (100%, 95%, 80%) before staining. The sections were stained with hematoxylin for 3–5 min, differentiated using 1% acid alcohol, and then blued with tap water. After rinsing, the sections were stained with eosin for 3–5 min, dehydrated through graded ethanol, cleared with xylene, and finally mounted with a coverslip for microscopic examination.

#### 2.2.6 Immunohistochemical staining

Procedures of deparaffinization and rehydration were the same as H&E staining. Antigen retrieval was performed by immersing sections in EDTA antigen retrieval solution (pH 9.0) and heating them in a microwave oven; after cooling at room temperature for 20 min, the sections were washed with PBS. To inhibit endogenous peroxidase activity, the sections were treated with 3% hydrogen peroxide for 25 min, followed by a 30-min incubation with 3% BSA in PBS to block non-specific binding at room temperature. The sections were then incubated with the primary antibody overnight at 4 °C in a humidified chamber, while negative controls were incubated with PBS. After washing with PBS, the sections were exposed to an HRP-labeled secondary antibody for 50 min at room temperature. The antigen-antibody complex was visualized using 3,3′-diaminobenzidine (DAB) as the substrate, resulting in a brown color at the site of the target antigen. Finally, the sections were lightly counterstained with hematoxylin for 3 min, dehydrated through graded ethanol, cleared in xylene, and mounted with a coverslip using mounting medium. The section was then examined using a microscope.

### 2.3 Target identification and verification of AUX-B

#### 2.3.1 Isolation and purification of AUX-B

The cultivation of fungal mycelia and the purification of metabolites are based on our previous research^27^. Briefly speaking, the metabolites of *A. umbrinum* were extracted with EtOAc. The resulting crude residue (20 g) was subjected to MCI gel column chromatography (solvent gradient: 20:80, 50:50, 70:3,0 and 100:0 CH_3_OH/H_2_O) to afford 6 subfractions (Frs. A–F). Fr. E was subjected to flash chromatography on an ODS column eluted with a gradient (0–20 min, 20% CH_3_OH/H_2_O; 20–40 min, 40% CH_3_OH/H_2_O; 40–90 min, 60% CH_3_OH/H_2_O; 90–120 min, 80% CH_3_OH/H_2_O; 120–150 min, 100% CH_3_OH/H_2_O) to give 12 subfractions (Frs. E1–E12). Frs. E8–E10 (783 mg) were combined and purified by semi-preparative HPLC (MeCN/H_2_O, 65:35) to obtain AUX-B (340 mg, *t*_R_ = 16.5 min). According to the above operation, a total of approximately 5.6 g AUX-B was obtained. The structure of AUX-B was confirmed by NMR and MS data (Supporting Information Figs. S1–S3, Table S3).

#### 2.3.2 Synthesis of AUX-B-p

AUX-B (400 mg, 1.2 mmol, 1 equiv), tetrabutylammonium bromide (TBAB) (39 mg, 0.12 mmol, 0.1 equiv), 5-iodo-1-pentyne (354 mg, 1.8 mmol, 1.5 equiv), and sodium hydroxide (NaOH) (73 mg, 1.8 mmol, 1.5 equiv) were dissolved in 2 mL of DMSO solvent. The reaction mixture was stirred at room temperature for 1 h, and then the reaction was terminated by adding one-fold deionized water. The mixture was extracted with 5 mL of ethyl acetate three times. The organic extracts were concentrated to dryness under vacuum. The residue was purified by semipreparative HPLC eluting with 68% (*v*/*v*) acetonitrile to yield AUX-B-p (196 mg, 35.9% yield). The chemical structure of AUX-B-p was determined by NMR and MS data (Supporting Information Figs. S4–S9, Table S3).

#### 2.3.3 Sample processing method based on ABPP

The U87 cells were incubated with 10 μmol/L of AUX-B-p probe in a cell incubator at 37 °C with 5% CO_2_ for 2 h. The cells were lysed using a probe sonicator in RIPA lysis buffer (C1053-100, Applygen, Beijing, China), which contained protease and phosphatase inhibitors. The resulting lysates were then centrifuged at 12,577 × *g* for 30 min at 4 °C.

#### 2.3.4 Chemical proteomics to discover the targets of AUX-B

Protein concentrations for each sample were normalized to 2 mg/mL with a volume of 1 mL. Click chemistry was performed on each sample using final concentrations of 5 μmol/L biotin azide and other reagents (CuSO_4_, Tris[(1-benzyl-1*H*-1,2,3-triazol-4-yl) methyl] amine (TBTA, GC45003-50, GLPBIO) and Tris(2-carboxyethyl) phosphine (TCEP, C4706, Sigma–Aldrich)) in a final volume of 1 mL for 1 h. After click chemistry, the proteins were then precipitated at 6500 × *g* for 5 min at 4 °C. Then, cold methanol was used to wash the precipitate sample, and the lysate was fractionated by centrifugation at 6500 × *g* for 5 min at 4 °C. The precipitated protein pellets were resuspended and dissolved in 1 mL of 1.2% SDS/PBS. The proteomes were boiled at 90 °C for 5 min and after centrifugation at 1400 × *g* for 1 min at room temperature, the supernatant was diluted to 0.2% SDS/PBS. The streptavidin beads (Pierce™ Streptavidin Agarose, 20353) were incubated with the supernatant for 3 h at room temperature. The beads were washed subsequently with PBS and deionized H_2_O for 3 times, respectively. The enriched proteins were digested by 2.5 μg trypsin in 200 μL of 2 mol/L urea/PBS with 2 μL of 100 mmol/L CaCl_2_ for 16 h at 37 °C. The digested peptide samples were desalted by Pierce™ C_18_ beforeior to MS analysis.

Proteomics was conducted using an LC‒MS/MS system composed of an Orbitrap Fusion™ Lumos™ Tribrid™ Mass Spectrometer (Thermo Fisher Scientific) coupled with an Ultimate 3000 LC system. Liquid chromatography separation was achieved on an analytical column (Acclaim PepMap RSLC 70 μm × 15 cm, nanoViper C18, 2 μm, 100 Å) at a flow rate of 300 mL/min using a 90-min gradient from 4% to 80% solvent B. The mobile phase was composed of water along with 0.1% formic acid (solvent A) and 80% acetonitrile along with 0.08% formic acid (solvent B). The peptides were ionized by using a spray voltage of 2.4 kV and a capillary temperature of 320 °C. The instrument was operated in data-dependent mode, automatically switching between MS and MS^2^ scans. For the full proteome samples, full-scan MS spectra (*m/z* 350–1500) were acquired with a maximum injection time of 50 ms at 60,000 resolution and an automatic gain control (AGC) target value of 4 × 10^5^ charges. High-resolution MS^2^ spectra were acquired with an exclusion duration of 25 s in the Orbitrap with a maximum injection time of 30 ms at 15,000 resolution (isolation window 1.6 *m/z*), an AGC target value of 5 × 10^4^ and normalized collision energy of 30%. Only precursors with charge states between 2 and 7 were selected for fragmentation.

#### 2.3.5 In-gel based ABPP

The sample processing method is consistent with the description in section 5.3.3. Click chemistry was performed on each sample using final concentrations of 100 μmol/L rhodamine azide and the above mentioned reagents in a final volume of 100 mL for 1 h. The reaction was quenched by cold acetone for 2 h at −20 °C. Next, centrifugation at 6500 × *g* for 10 min at 4 °C to obtain the labeled protein, followed by the addition of 45 mL 1 × SDS loading buffer. The samples were then loaded (20 mL) and resolved on a 10% SDS-PAGE gel. The gels were scanned by fluorescence (Typhoon FLA9500, Ettan DIGE, United States) and stained by Coomassie brilliant blue to demonstrate equal loading.

#### 2.3.6 Expression of recombinant SCAMP2 truncation, mutant, and full-length protein

Recombinant SCAMP2 truncation (residues 1–151) and mutant (K130A, C127A, C132A, C149A, C127/132A) plasmids were purchased from Youbio Biological Technology Co., Ltd., Changsha, China. Then, plasmids were transformed into *E. coli* strain BL21 (DE3) (CD601-03, TransGen Biotech Co., Ltd., Beijing, China), grown until OD_600_ of 0.5, then incubated with 1 mmol/L IPTG (isopropyl-D-thiogalactoside) at 16 °C for 18 h, and harvested. Lysate was incubated with Ni-IDA-Sepharose before being removed of nonspecific adsorption by washing with a suitable buffer, after concentration and desalting to obtain recombinant SCAMP2 truncation and mutant. SCAMP2 full-length protein was purchased from OriGene Co., Ltd., Beijing, China (TP300262M).

#### 2.3.7 SPR assay

The binding affinities of AUX-B with its target proteins, his-SCAMP2 (full-length and truncated), were assayed by using the SPR-based Biacore X 100 instrument (Cytiva, France). The CM5 sensor chip was used to immobilize 10,000–15,000 RU of the target protein to the sensor surface by the standard amine coupling reaction at 25 °C in PBS running buffer. Gradient concentrations of AUX-B containing 5% DMSO were injected into the channels to evaluate the binding affinity. The analysis was conducted using the Biacore X 100 Evaluation Software.

#### 2.3.8 Identification of the AUX-B-binding site on SCAMP2 truncation by a filter-aided sample preparation (FASP)

Recombinant SCAMP2 truncation protein (50 mL, 10 mmol/L) was incubated with AUX-B (1 mL, 10 mmol/L) for 2 h at room temperature. The resulting mixture was then added with 100 μL of 8 mol/L urea and 100 mmol/L DTT, and was then incubated at 37 °C for 1 h. Next, the sample was transferred to a 10-kDa cut-off filter (Microcon-10 kDa Centrifugal Filter Unit with Ultracel-30 membrane, Millipore). Centrifugating at 14,000 × *g* for 15 min, the sample was washed with 100 μL of 50 mmol/L NH_4_HCO_3_ at 14,000 × *g* for 15 min. Then 100 μL 20 mmol/L IAA was added to the filter, followed by further incubation for 1 h in the dark. After centrifugation at 14,000 × *g* for 15 min, the sample was washed with 100 μL of 50 mmol/L NH_4_HCO_3_ three times. Next, 150 μL of 10 mmol/L NH_4_HCO_3_ and trypsin (trypsin: protein = 1:100, *w/w*) was added to the sample, followed by further incubation at 37 °C for 16 h. The resulting peptides were extracted and desalted for downstream LC‒MS/MS analysis (conditions and analysis are consistent with those for chemical proteomics).

### 2.4 Multi-omics analysis

#### 2.4.1 Targeted metabolomics analysis in GBM cells

GBM cells U87 and U251 treatment with AUX-B for 24 h or U87 cells after knockout and overexpression of SCAMP2, were quenched and extracted by 1.4 mL of ice-cold 80% MeOH for 30 min at 4 °C, followed by centrifugation and supernatant drying. After reconstitution with 0.5 mL 70% ACN, a Nexera LC-30A UHPLC system (Shimadzu, Kyoto, Japan) equipped with a Waters BEH Amide column (2.1 mm × 100 mm, 1.7 μm, Waters, Milford, MA, USA) was used for the metabolites separation. Mobile phase A was 10 mmol/L ammonium acetate and 0.2% acetic acid in 10% ACN, while mobile phase B was 90% ACN. The column was maintained at 40 °C, and the flow rate was 0.3 mL/min. The mobile phase gradient was set as follows: 1.5 min, 90% of B; 5 min, 45% of B; 10 min, 45% of B; 12 min, 90% of B; 25 min, 90% of B. Mass spectroscopic (MS) analyses were performed by using an electrospray ionization (ESI) probe. The source parameters were as follows: Curtain Gas, 35; Collision Gas, Medium; IonSpray Voltage, 5.5 kV/–4.5 kV; temperature, 400 °C; Ion Source Gas 1, 55 Psi; Ion Source Gas 2, 55 Psi. The multiple reaction monitoring (MRM) mode was used to detect metabolites of interest according to our previously published protocol^31^. A total of 236 m 40 metabolic pathways were selected for targeted analysis. Data were acquired using Analyst 1.7.2 software and analyzed using SCIEX OS 2.1.6.59781 software (AB Sciex, Framingham, MA).

#### 2.4.2 Cellular metabolic flux

U87 cells with or without overexpression of SCAMP2 or not were divided randomly into three groups with 4 duplicates, including OENC and OESC2. Then, cells were cultured in DMEM (addition of external ^13^C, ^15^N-aspartate or replacing the original ^12^C-glutamine with ^13^C-glutamine) for 24 h. Metabolites in cells were then extracted by 1.4 mL of a cold 80% MeOH, followed by centrifugation and supernatant drying. After reconstitution with 0.5 mL 70%ACN, a LC–MS system composed of a Vanquish Flex System coupled to a high-resolution quadrupole-orbitrap tandem mass spectrometer (Q-Exactive plus; Thermo Fisher Scientific, Waltham, MA, USA) was used to detect metabolic flux. Liquid chromatography separation was achieved on an ACQUITY UPLC BEH Amide column (100 mm × 2.1 mm, 1.7 μm particle size, Waters, Milford, MA, USA) under the following chromatographic conditions: mobile phase A (water: acetonitrile, 95:5, 5 mmol/L ammonium acetate, 0.1% acetic acid) and mobile phase B (water: acetonitrile, 5:95, 5 mmol/L ammonium acetate, 0.1% acetic acid) at a flow rate of 0.3 mL/min and column oven temperature at 35 °C. The gradient started with 94% B and was held for 1 min. It decreased to 78% B over 6.5 min, then decreased to 39% B over 4.5 min, and held at 39% B for 5 min before further increasing to 94% B over 2 min. The gradient was finally equilibrated for 6 min. The autosampler temperature was 10 °C, and the injection volume was 2 μL and 10 μL under positive and negative ion modes, respectively. Data-dependent MS^2^ assays comprised a full scan (*m/z* 70–1000; resolution, 70,000; AGC target, 3e6; maximum IT, 100 ms) and an MS/MS scan (resolution, 17,500; AGC target, 1e5; and maximum IT, 50 ms; loop count, 15; NCE, 20,40,60). The ESI source parameters were as follows: the capillary temperature was 320 °C/350 °C, sheath gas 35, aux gas 15, auxiliary gas heater temperature 350 °C/300 °C, spray voltage 3.5 kV/–3.0 kV.

#### 2.4.3 Proteomics analysis in GBM cells

GBM cells U87 and U251 treated with AUX-B or not for 24 h were homogenized in 600 μL of T-PER tissue protein extraction reagent (Thermo Fisher Scientific, 78510) containing a protease inhibitor, and incubated with vortexing at 4 °C for 30 min. The mixture was homogenized again and incubated at 4 °C for 20 min. The supernatant was retained after centrifuging for 1 min (8000 × *g*, 4 °C). Subsequently, the sample was sonicated at 4 °C for 10 s on and 10 s off with a total working time of 30 min. The insoluble debris was removed by centrifugation at 16,000 × *g* for 20 min. The supernatant was retained and quantified by the bicinchoninic acid (BCA) assay. The extracted proteins (50 μg) from each sample were diluted to 200 μL with 50 mmol/L ammonium bicarbonate. Proteins were reduced and alkylated with dithiothreitol (DTT, Sigma, 1 mol/L, 56 °C, 1 h) and iodoacetamide (IAM, Sigma, 1 mol/L, 25 °C in the dark, 30 min), respectively. The pre-cooled acetone (1 mL) was added and precipitated overnight at −30 °C. After centrifugation, the supernatant was removed. The precipitate was washed with 500 μL pre-cooled acetone, centrifuged again, and dried in a fume hood. Proteins were digested with 1 μg of sequence-grade trypsin (Promega, V5117, dissolved in 50 mmol/L ammonium bicarbonate, 37 °C, 16 h). The reaction was quenched with 10% of trifluoroacetic acid, then 20 μL of the peptides were desalted, dried, resuspended, and added to iRT before LC–MS analysis.

Proteomics was conducted using an LC–MS system composed of a nanoflow Easy-nLC 1200 system coupled to an Orbitrap Exploris 480 mass spectrometer (Thermo Fisher Scientific, Waltham, MA, USA). Liquid chromatography separation was achieved on an in-house packed capillary column (75 μm i.d. × 25 cm, ReproSil-Pur C18-AQ, 1.9 μm; Dr. Maisch). The mobile phase was composed of water along with 0.1% formic acid (A) and 80% acetonitrile along with 0.1% formic acid (B). LC gradient: 0−2 min, 3%−8% buffer B; 2−54 min, 8%−28% buffer B; 54−70 min, 28%−100% buffer B; 70−78 min, 100% buffer B. The temperature of the column was 55 °C, and the flow rate was 300 nL/min. The MS analysis was operated in the data-independent (DIA) mode. The spray voltage was set at 2100 V, and the capillary temperature was set at 320 °C. The resolution and maximum injection time MS1 were set at 60,000 and 50 ms. Sixty DIA windows scanned from *m/z* 350 to 1500 with a resolution of 15,000, where precursor ions were fragmented with a normalized collision energy of 30%.

### 2.5 Data and statistical analysis

For the targeted metabolomic and the metabolic flux, peak integration of AUX-B and all targeted metabolites was achieved using the SCIEX OS software (version 2.1.6.59781). The distribution of the coefficients of variation (CV) of all the measured metabolites in the QC samples was calculated. Metabolites with CV < 20% in the QC samples and missing values < 50% in all the samples were used for the next analysis. The metabolite data were normalized with the mean of adjacent QCs and further normalized by the protein concentration of each sample. Peak integration of flux data was performed using the TraceFinder™ 5.1 software. The isotope ratio was corrected by natural isotope abundances using a customized R script, which was written by adapting the AccuCor algorithm.

For the MSI, the original data were converted to.cdf format using Xcalibur software before background subtraction and image reconstruction using MassImager software. For the proteomics, the data were analyzed using Spectronaut 18.6.231227.55695 (Biognosys), and MS/MS spectra were searched against the UniProt human proteome database (20424 entries, 2023/09/12). For the chemical proteomic, LC‒MS/MS data were analyzed by MaxQuant software (1.6.5.0) and default settings with static modification of cysteine (+57.0215 Da) and variable oxidation of methionine (+15.9949 Da). The resulting data were further analyzed by using the Perseus software (v1.6.15.0). The default settings were used unless otherwise described. The false discovery rates of peptides and proteins were all <0.01. For the LC–MS/MS-based identification of the AUX-B-binding site on SCAMP2, precursor mass tolerance was set to 10 ppm, and fragment mass tolerance was 0.6 Da.

Data are expressed as mean ± standard deviation (SD). To determine statistical significance between two groups or among multiple groups, an unpaired Student’s *t*-test, one-way ANOVA, or two-way ANOVA was employed, respectively. Statistical analysis was conducted using GraphPad Prism 10 (GraphPad Software Inc., San Diego, CA, USA), with a *P*-value of less than 0.05 deemed statistically significant.

## 3 Results

### 3.1 AUX-B demonstrated anti-glioblastoma activity both *in vitro* and *in vivo*

**Figure 1:**
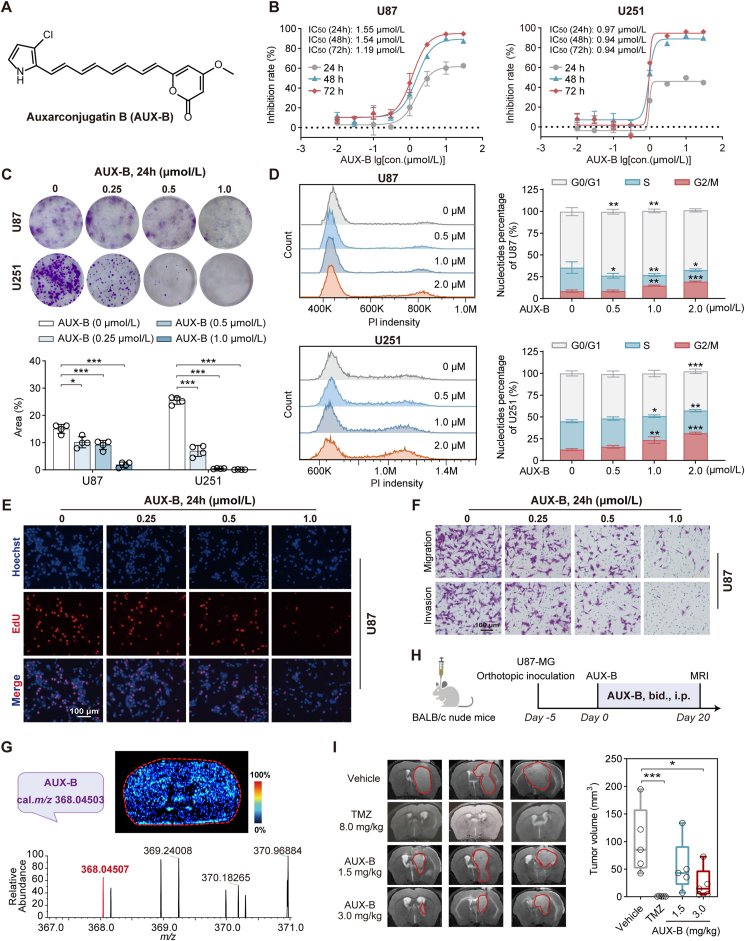
AUX-B demonstrated anti-glioblastoma activity both *in vitro* and *in vivo*. (A) The chemical structure of Auxarconjugatin B (AUX-B). (B) Cell viability of U87 (left) and U251 (right) was determined by cell counting kit-8 (CCK-8) assay after incubation with 0, 0.01, 0.03, 0.1, 0.3, 1.0, 3.0, 10, and 30 μmol/L AUX-B for 24, 48, and 72 h. IC_50_ at 24, 48, and 72 h was calculated and noted, respectively. (C) The macrographs of plate colonies in U87 and U251 cells treated with 0, 0.25, 0.5, and 1.0 μmol/L AUX-B (*n* = 4). (D) Analysis of cell cycle phase distribution was conducted by flow cytometry using propidium iodide staining following treatment with 0, 0.5, 1.0, and 2.0 μmol/L AUX-B in U87 (top) and U251 (bottom) cells, respectively. (E) The DNA synthesis rate was measured by DNA-EdU assay following treatment with 0, 0.25, 0.5, and 1.0 μmol/L AUX-B for 24 h in U87 cells. (F) AUX-B inhibited the migration and invasion of U87 cells by the transwell assay. (G) Mass spectrometry image (MSI) of AUX-B in the murine brain after the intraperitoneal administration of AUX-B (15 mg/kg) and sacrificed at 30 min (Intensity in MSI color scale is a relative value. AFADESI-MSI experiments were performed three times independently from different animals with similar results. Resolution, 100 μm). The bottom spectrum demonstrated the quasi-molecular ion peak of AUX-B with a K^+^ adduct in high-resolution MS. (H) Schematic outlines of the construction of U87-derived orthotopic tumor model in BALB/c nude mice. The mice were treated with either TMZ (8 mg/kg once daily i.g.), AUX-B (1.5 mg/kg or 3.0 mg/kg twice daily i.p. injection), or a solvent (twice daily, i.p. injection) as the vehicle group. (I) Representative magnetic resonance imaging of the vehicle and treatment groups with TMZ (8.0 mg/kg) or AUX-B (1.5 or 3.0 mg/kg) in the U87-derived orthotopic model. The tumor volumes with different treatments were calculated in the orthotopic model (*n* = 5). Data are given as mean ± SD, ∗*P* < 0.05, ∗∗*P* < 0.01, and ∗∗∗*P* < 0.001, analyzed by one-way ANOVA. M, mol/L.

During our investigation of the biosynthesis of rumbrins, a group of polyenylpyrrole-type polyketides, dozens of rumbrin analogs were obtained^27^. AUX-B (Fig. 1A), one of the rumbin analogs with a conjugated polyene structure and balanced hydrophobicity, demonstrates a low IC_50_ value against multiple tumor cell lines^32^^,^^33^. We therefore evaluated the effect of AUX-B on cell viability and proliferation in GBM cell lines U87 and U251. The CCK-8 assay demonstrated that AUX-B inhibited the growth of U87 and U251 cells in a time- and dose-dependent manner (Fig. 1B). Specifically, the IC_50_ values for U87 cells at 24, 48, and 72 h were 1.55, 1.54, and 1.19 μmol/L, respectively. For U251 cells, the corresponding IC_50_ values were 0.97, 0.94, and 0.94 μmol/L. AUX-B was also found to significantly inhibit colony formation in a dose-dependent manner (Fig. 1C) and arrest the cell cycle at the G2/M phase (Fig. 1D) in both U87 and U251 cells. The results from the EdU assay of U87 and U251 cells showed that AUX-B remarkably suppressed DNA synthesis in a dose-dependent manner at 24 and 48 h (Fig. 1E and Supporting Information Fig. S10A–S10C). Further Transwell assay demonstrated that AUX-B significantly reduced the migration and invasion of U87 and U251 cells (Fig. 1F, Fig. S10D and S10E). In addition, toxicity screening revealed that the toxic effect of AUX-B on normal cells was not as strong as that on tumor cells (Fig. S10F). Collectively, AUX-B demonstrated anti-GBM activity *in vitro*.

The blood–brain barrier (BBB) is recognized as one of the biggest obstacles in the development of anti-GBM drugs^34^. Therefore, we further performed brain penetration studies of AUX-B in mice. As shown in Fig. 1G, the quasi-molecular ion at *m/z* 368.04503 assigned to a K^+^ adduct with AUX-B, was extensively distributed throughout the brain after intraperitoneal administration of AUX-B at a dose of 15 mg/kg by air-flow assisted desorption electrospray ionization mass spectrometry imaging. These results suggested that AUX-B may cross into the brain, which is consistent with the results predicted from the ADME websites and derived from tissue distributions of AUX-B after intraperitoneal administration (Supporting Information Fig. S11A–S11D). To assess the *in vivo* anti-GBM activity of AUX-B, we established an orthotopic and subcutaneous xenograft GBM mouse model by injecting U87 cells, respectively. After 5 or 14 days, tumor-bearing mice were administered with vehicle or various doses of AUX-B *via* intraperitoneal injection (Fig. 1H and Fig. S11E). AUX-B at a dose of 3.0 mg/kg significantly suppressed the growth of GBM xenograft tumors, with an obvious reduction in the tumor volume and weight as compared to the vehicle group (Fig. 1I, Fig. S11F and S11G). In addition, there was no significant difference in the body weight, hematoxylin–eosin (H&E) staining of various organs, and organ weight ratio (Fig. S11H–S11J) between the vehicle group and the AUX-B-treated groups in the U87 cells-derived orthotopic GBM mice model. Notably, AUX-B was also detectable *via* LC–MS/MS in both brain and tumor tissues at this therapeutic dose, where the concentrations detected in brain and tumor tissues were approximately 0.13–0.24 ng/mg (brain) and 0.14–0.20 ng/mg (tumor), respectively (Fig. S11K and S11L). Taken together, these findings suggested that AUX-B has the potential to inhibit GBM growth without noticeable side effects at a dosage of 3.0 mg/kg.

### 3.2 AUX-B directly targets SCAMP2 in GBM cells

**Figure 2:**
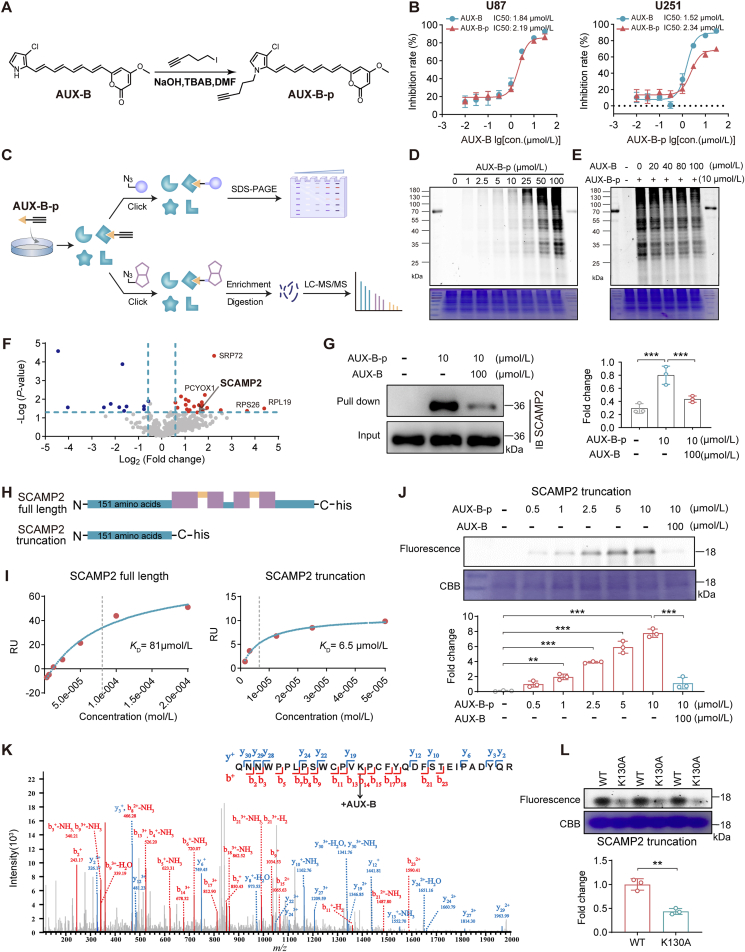
Identification of SCAMP2 as a direct target of AUX-B. (A) Chemical probe AUX-B-p was synthesized from AUX-B and 5-iodo-1-pentyne. (B) A CCK-8 assay was used to analyze the effect of AUX-B and AUX-B-p on U87 cells (left) and U251 (right) cells viability, respectively. Cells were treated with various doses of AUX-B and AUX-B-p for 24 h. (C) Schematic illustration of the click chemistry based small-molecule probe for AUX-B target labelling and pull-down. The diagram outlines the activity-based protein profiling strategy for identifying targets of small molecule probes in live cells. U87 cells treated with a probe containing an alkyne fragment were subjected to click reactions with rhodamine (top) or biotin (bottom) azide-containing fragments. The rhodamine-labeled proteome was separated by SDS-PAGE and visualized by fluorescence in-gel scanning, while the biotin-labeled proteome was enriched using streptavidin beads and subsequently analyzed by mass spectrometry. (D) In-gel fluorescence scanning was used to exhibit the labelled proteomes. Concentration-dependent activity-based protein profiling labeling of U87 cells with AUX-B-p probe. (E) Competitive labeling of AUX-B-p probe with AUX-B in U87 cells. (F) Volcano plot from gel-free quantitative LC‒MS experiments comparing proteomes labeled with AUX-B-p (10 μmol/L) *versus* DMSO (negative control). The plot highlights significantly down-regulated proteins (blue), up-regulated proteins (red), and non-significantly altered proteins (grey). The *x*-axis represents the log_2_ fold change in protein expression, while the *y*-axis indicates statistical significance (–log_10_*P*-value). Data are from four independent replicates (*n* = 4). (G) Pull-down Western blot analysis was performed to identify SCAMP2 protein labeled by the AUX-B-p probe and to evaluate the competitive inhibition effect of AUX-B (*n* = 3). The labeled proteomes were “clicked” with a biotin-azide reporter (“input”), pulled down by avidin beads, and eluted by boiling the beads (“output”). The input and output samples were analyzed by anti-SCAMP2 immunoblotting. (H) The schematic diagram of SCAMP2 and its truncations. The blue color represents the region inside the membrane, the yellow color represents the region outside the membrane, and the purple color represents the transmembrane region. (I) Surface plasmon resonance analysis of AUX-B binding to SCAMP2 (left) and SCAMP2 truncations (right), individually. (J) Concentration-dependent labeling of recombinant SCAMP2 truncation by AUX-B-p and competitive inhibition using various concentrations of AUX-B (*n* = 3). (K) Representative MS/MS site-mapping data showing the identification of Lys130 as the main reacting site of AUX-B in recombinant SCAMP2 truncation in a MS^2^ spectrum of the peptide with *m/z* 4055.7975 Da. (L) The fluorescence labeling of recombinant SCAMP2 truncation and mutant K130A by AUX-B-p (*n* = 3). Data are given as mean ± SD, ∗*P* < 0.05, ∗∗*P* < 0.01, and ∗∗∗*P* < 0.001, analyzed by Student’s *t*-test or one-way ANOVA.

The prominent inhibitory effects of AUX-B on GBM *in vitro* and *in vivo* prompted us to identify its possible targets. A terminal alkyne-containing clickable molecular probe, designated AUX-B-p (Table S3), was synthesized from a nucleophilic substitution reaction (Fig. 2A) to selectively enrich probe-bound proteins from live cells, using an activity-based protein profiling strategy. The comparable bioactivity of AUX-B-p to AUX-B was first evidenced by its inhibitory effects on U87 and U251 cell proliferation (Fig. 2B). Following the labeling of AUX-B-p with proteins in U87 cell lysate, the labeled proteins were conjugated with rhodamine-azide for subsequent separation by SDS-PAGE, followed by detection *via* in-gel fluorescence scanning (Fig. 2C). Visualization of the enriched proteins revealed prominent bands at approximately 30–36 kDa and 40–55 kDa, with band intensities increasing in a manner dependent on both probe concentration (Fig. 2D) and incubation time (Supporting Information Fig. S12A). In competitive binding assays, pre-treatment with AUX-B markedly reduced the intensity of these bands, suggesting that the corresponding proteins may serve as potential targets of AUX-B (Fig. 2E). Furthermore, the AUX-B-p labeled proteins were conjugated with a biotin-azide reporter to facilitate the enrichment and subsequent identification *via* mass spectrometry (Fig. 2C). A total of 22 proteins exhibiting a fold change >1.5 and *P*-value <0.05 were successfully identified (Fig. 2F, Supporting Information Table S4). Candidate proteins were selected from this set for subsequent validation, based on two criteria: 1) molecular weights within the 30–36 kDa or 40–55 kDa range (consistent with the observed band sizes), and 2) higher fold changes or more significant *P*-values. Of these candidates, only SCAMP2 was successfully pulled down by the probe; notably, this pull-down interaction was abrogated by pre-treatment with AUX-B. A follow-up pull-down assay further demonstrated that AUX-B-p effectively captured native SCAMP2 in U87 cells, and this capture effect was significantly abolished when samples were pre-treated with AUX-B (Fig. 2G). These results initially indicated that AUX-B directly targets SCAMP2 in U87 cells.

Next, we investigate the interaction and binding sites of AUX-B with SCAMP2. Structurally, SCAMP2 typically contains four transmembrane domains that anchor it within the membrane as well as cytoplasmic N- and C-terminal regions that interact with other cellular components (Fig. 2H)^35^. The 151 amino acids flexible peptide located on the N-terminal domains might play a crucial role in the structure and function of SCAMP2, and is preferred as a truncation first. Hence, both the truncated SCAMP2 and its full-length protein were recombinantly expressed. By surface plasmon resonance (SPR) assay, we found that AUX-B could interact with both full-length and truncated SCAMP2 (Fig. 2I), indicating AUX-B bound to the truncated peptide (residues 1–151). This result was further demonstrated by AUX-B-p labeling SCAMP2 truncation and competitive inhibition by AUX-B (Fig. 2J). Subsequently, we investigated the direct binding site of AUX-B within SCAMP2. LC‒MS/MS analysis of the SCAMP2 truncation–AUX-B complex revealed that AUX-B covalently modified a specific peptide of SCAMP2 (spanning residues 117–147) with the modification occurring at lysine (K) 130 (Fig. 2K). To further determine whether this residue is critical for the binding process, a recombinant K130A mutant of the SCAMP2 truncation was prepared and labeled with AUX-B-p. A significant reduction in binding was observed compared to the wild-type SCAMP2 truncation (Fig. 2L). Given that the presence of an *α*,*β*-unsaturated carbonyl group in AUX-B makes it potentially covalently bind to the cysteine (C) of SCAMP2 truncation through a Michael addition reaction, the C127A, C132A, and C149A mutants were also obtained (Fig. S12B). There were no significant alterations between AUX-B-p labeling C-mutants and wild-type SCAMP2 truncation (Fig. S12C), since the possibility of AUX-B binding to cysteine residues in the truncation was excluded. This finding was further validated by microscale thermophoresis (MST) assays (Fig. S12D), where AUX-B interacted with the recombinant truncated SCAMP2 wild type (*K*_D_ = 0.35 μmol/L) but not with the K130A mutant. This further confirms that Lys130 is the key binding site for AUX-B to SCAMP2. Together with the results of the decreased protein expressions (Fig. S12E and S12F) and inconsistent mRNA expression changed levels (Fig. S12G and S12H) regulated by AUX-B in U87 cells, it is suggested that AUX-B covalently binds to SCAMP2 and induces protein reduction rather than transcription inhibition.

### 3.3 SCAMP2 was highly expressed in GBM tumor tissues and promoted tumor proliferation

**Figure 3:**
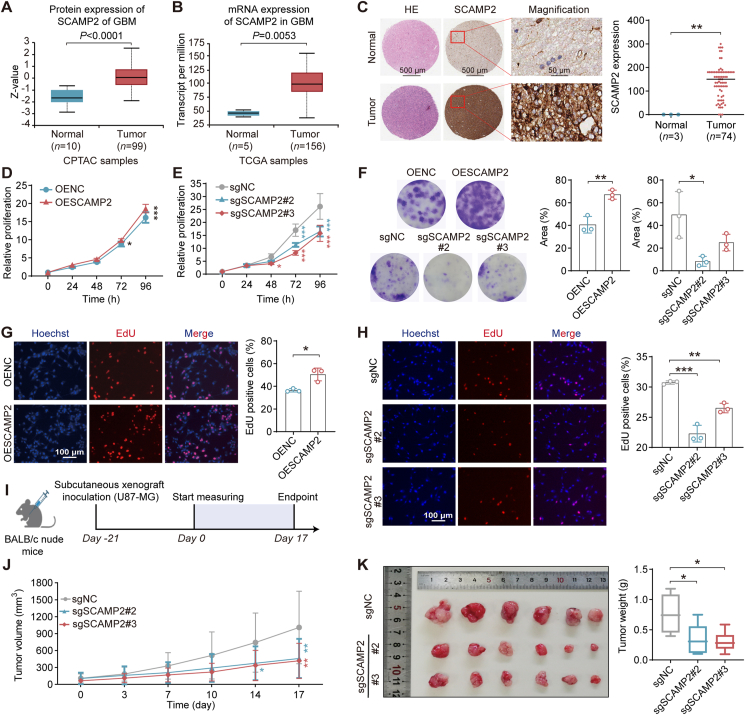
SCAMP2 is highly expressed in GBM tissues and promotes tumor proliferation. (A) Protein expression analysis of SCAMP2 in normal (*n* = 10) and primary tumors (*n* = 99) from the GBM datasets of Clinical Proteomic Tumor Analysis Consortium was performed using the UALCAN portal. (B) The mRNA expression analysis of SCAMP2 in normal (*n* = 5) and primary tumors (*n* = 156) from the GBM datasets of The Cancer Genome Atlas was performed using the UALCAN portal. (C) SCAMP2 expression in GBM patients revealed by immunohistochemistry staining from a tissue microarray is shown in the normal group (*n* = 3) and tumor group (*n* = 74). The left picture represents hematoxylin-eosin staining, and the middle picture represents immunohistochemistry staining (scale bar: 500 μm). The right pictures are the enlarged representations of the boxed regions of the middle pictures (scale bar: 50 μm). (D) CCK-8 assays demonstrated that overexpression of SCAMP2 (OESCAMP2) increased the viability of U87 cells. OENC, overexpression negative control. (E) CCK-8 assays demonstrated that depletion of SCAMP2 (sgSCAMP2#2 and sgSCAMP2#3) reduced the viability of U87 cells. sgSCAMP2#2 and sgSCAMP2#3, knockout SCAMP2 with 2 different sgRNAs by CRISPR/Cas9 technology; sgNC, sgRNA negative control. (F) The macrographs of plate colony formation in U87 cells with SCAMP2 overexpression (OESCAMP2) and knockout (sgSCAMP2#2 and sgSCAMP2#3). A DNA-EdU assay was used to measure the DNA synthesis rate in U87 cells with SCAMP2 overexpression (G) and knockout (H), respectively. (I) Schematic diagram of tumor inoculation and measurement protocol in U87-derived subcutaneous tumor mouse models, the tumor volume was measured on the 21st day after subcutaneous injection of cells, and terminated in 17 days. (J) Tumor growth curves of sgNC and SCAMP2-knockout U87-derived subcutaneous xenograft tumor mouse models (*n* = 6). (K) Images of tumors in sgNC and SCAMP2-knockout U87-derived subcutaneous xenograft tumor mouse models, and the tumor weight of the subcutaneous xenograft tumor was calculated in each group (*n* = 6). Data are given as mean ± SD, ∗*P* < 0.05, ∗∗*P* < 0.01, and ∗∗∗*P* < 0.001, analyzed by Student’s *t*-test, one-way ANOVA, or two-way ANOVA.

To determine whether SCAMP2 promotes GBM proliferation, we initially investigated the expression level of SCAMP2 in human GBM tissues using UALCAN, an integrated cancer data analysis platform^36^. It was shown that significantly higher protein (Fig. 3A) and mRNA expression levels (Fig. 3B) of SCAMP2 were observed in GBM tissues compared to normal tissues. We assessed SCAMP2 protein expression in GBM tissue microarrays, including 74 human GBM tissues and 3 adjacent normal tissues. Immunohistochemistry (IHC) staining revealed significantly higher SCAMP2 levels in GBM tissues compared to normal tissues (Fig. 3C). These observations underscore the clinical significance of SCAMP2 in GBM. Furthermore, elevated protein expression of SCAMP2 was found in tumor tissues of the GL261-derived orthotopic GBM model by IHC analysis (Supporting Information Fig. S13A). Collectively, these data revealed that SCAMP2 is highly expressed in GBM tumor tissues and is regarded as a potential therapeutic target.

To provide direct evidence for the role of SCAMP2 in GBM, we conducted knockout (Fig. S13B) and overexpression (Fig. S13C) of *SCAMP2* in U87 cells, respectively. It was found that overexpression of *SCAMP2* resulted in the promotion of U87 cell proliferation (Fig. 3D) while deletion of *SCAMP2* decreased cell viability by CCK-8 assay (Fig. 3E). Overexpression and deletion of *SCAMP2* also significantly promoted and inhibited the colony formation in U87 cells, respectively (Fig. 3F). In addition, an EdU-DNA synthesis assay confirmed that overexpressed *SCAMP2* dramatically promoted the proportion of the replicating U87 cells population, and is opposite for *SCAMP2* knockout (Fig. 3G and H). In addition, overexpression of SCAMP2 in U87 cells stimulated their proliferation and significantly rescued AUX-B-induced cell death (Fig. S13D). In contrast, U87 cells with the knockout of SCAMP2 seem more sensitive to AUX-B (Fig. S13E), suggesting that AUX-B had minimal off-target effects. Next, we tested the inhibitory effect of genetic deletion of *SCAMP2* on the ability of tumorigenicity by subcutaneously injecting *SCAMP2*-deleted U87 cells into BALB/c nude mice (Fig. 3I), which resulted in significant suppression of tumor volume and weight in comparison to the control group and without affecting body weight (Fig. 3J and K, Fig. S13F). Thus, these findings support the conclusion that *SCAMP2* is an oncogene that promotes sustained proliferation in U87 cells.

### 3.4 Mediating SCAMP2 by AUX-B induced aspartate metabolic dysfunction

**Figure 4:**
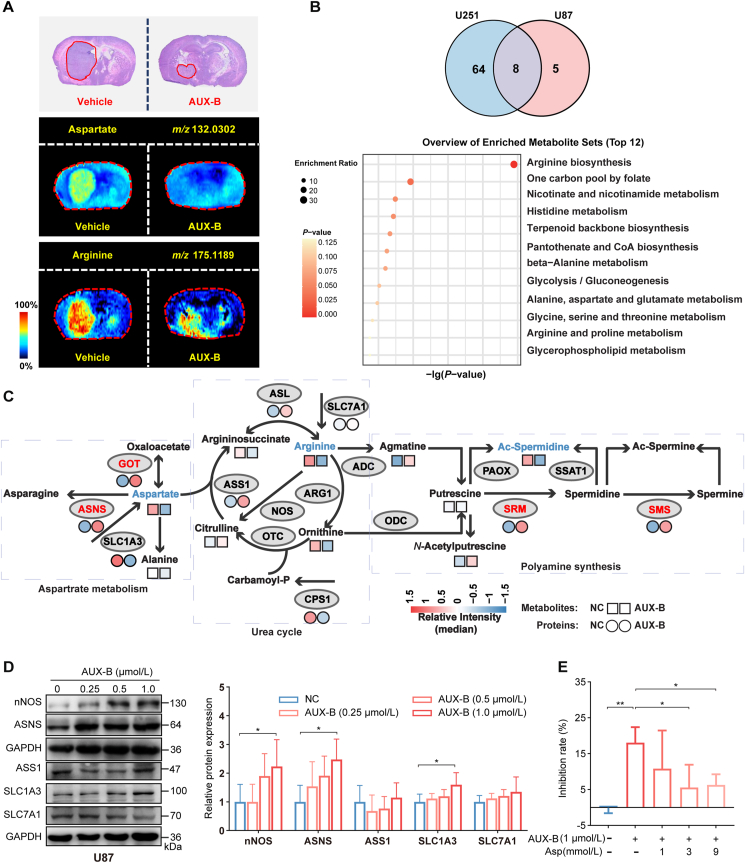
Reduction of SCAMP2 by AUX-B induced aspartate metabolic dysfunction. (A) Representative hematoxylin-eosin staining images of brain tissues collected from U87-derived orthotopic tumor models with or without AUX-B treatment. MSI showed the representative aspartate (middle) and arginine (bottom) distribution and relative abundance in coronal section of the brain (*n* = 3). (B) Venn diagram displayed 8 overlapped objects (FC > 1.5 or < 0.67 and *P* < 0.05 for AUX-B vs. vehicle in U87; FC > 1.5 or <0.67 and *P* < 0.05 for AUX-B vs. vehicle in U251) between two groups (top). Top 12 pathways of KEGG enrichment analysis by 8 differential metabolites in U87 and U251 cells are displayed in a bubble chart (bottom). The diameter of the circle is indicative of the corresponding enrichment ratio, while the intensity of the red color reflects the magnitude of the *P*-value. (C) Schematic representation of aspartate–arginine metabolism. Circles below enzymes and boxes below metabolites indicate changes in protein and metabolite levels in U87 cells treated with (right) or without (left) 1 μmol/L AUX-B, respectively. Color coding is based on level of log_2_-fold change as indicated (*n* = 4). NOS, nitric oxide synthase; ASNS, asparagine synthetase; ASS1, argininosuccinate synthetase 1; SLC1A3, solute carrier family 1 member 3; SLC7A1, solute carrier family 7 member 1. (D) Western blot analysis of enzymes and transporters related to aspartate–arginine metabolism in U87 cells treated with various doses of AUX-B and their quantitation (*n* > 3). (E) Aspartate supplementation experiment. U87 cells were treated with 1 μmol/L AUX-B or left untreated, and simultaneously were supplemented with aspartate at 1, 3, and 9 mmol/L or without aspartate supplementation for 24 h. Subsequently, a CCK-8 assay was employed to evaluate the viability of U87 cells. Data are given as mean ± SD, ∗*P* < 0.05, ∗∗*P* < 0.01 and ∗∗∗*P* < 0.001, analyzed by Student’s *t*-test or one-way ANOVA.

Although glutamate metabolism significantly affects GBM proliferation^37^, the metabolic reprogramming induced by other amino acids in GBM proliferation remains poorly understood. We first noted that both aspartate and arginine levels were significantly higher in GBM tissue compared to paracancerous brain tissue in the orthotopic tumor model by untargeted metabolomics using MSI (Fig. 4A, Supporting Information Table S5). Among these, the aspartate level was significantly restored following AUX-B administration. To further explain the cellular metabolic landscape that is regulated by SCAMP2, a targeted metabolomic analysis was performed on AUX-B-treated U87 and U251 cells according to our previous study^38^. Altogether, 72 and 13 significantly different metabolites (Supporting Information Table S6) were found in U251 cells and U87 cells, respectively (Fig. 4B). Further, KEGG enrichment analysis of the 8 overlapping differential metabolites indicated that arginine metabolism was the most impacted metabolic pathway by SCAMP2 inhibition.

Since aspartate is involved in arginine synthesis, a detailed analysis of metabolites in arginine metabolism was performed. As shown in Fig. 4C, three arginine-related metabolic pathways are displayed: aspartate metabolism, the urea cycle, and polyamine metabolism. Notably, AUX-B treatment resulted in significant decreases in aspartate, arginine, and acetyl-spermidine levels in U87 cells (Supporting Information Fig. S14A) and marked reduction in aspartate, arginosuccinate, and asparagine levels in U251 cells (Fig. S14B). The proteomics analysis revealed that the metabolic enzymes associated with aspartate synthesis and utilization, such as glutamic-oxaloacetic transaminase 1, ASNS, arginosuccinate synthase, and SLC1A3, exhibited varying degrees of alteration in U87 and U251 cells (Fig. 4C, Supporting Information Fig. S14C and Table S7). Among them, the levels of ASNS are negatively correlated with those of SCAMP2 (correlation coefficient *R* = −0.7388), while the expression of the mitochondrial aspartate shuttle SLC25A12 is positively correlated with SCAMP2 (*R* = 0.7243) (Fig. S14D). Further detection in these metabolic enzymes indicated that targeting SCAMP2 by AUX-B leads to a simultaneous elevation in mRNA and protein expressions of ASNS in these two cells (Fig. 4D and Fig. S14E–S14G). These results suggest that modulating SCAMP2 by AUX-B leads to increased utilization of aspartate and a concomitant decrease in aspartate levels. In fact, a rescue experiment confirmed that replenishing aspartate reduced the efficacy of AUX-B (Fig. 4E). Hence, the increased aspartate regulated by SCAMP2 is essential for the growth of GBM cells, which could be modulated by AUX-B.

### 3.5 SCAMP2 reprogrammed aspartate metabolism mainly through the aspartate transporter

**Figure 5:**
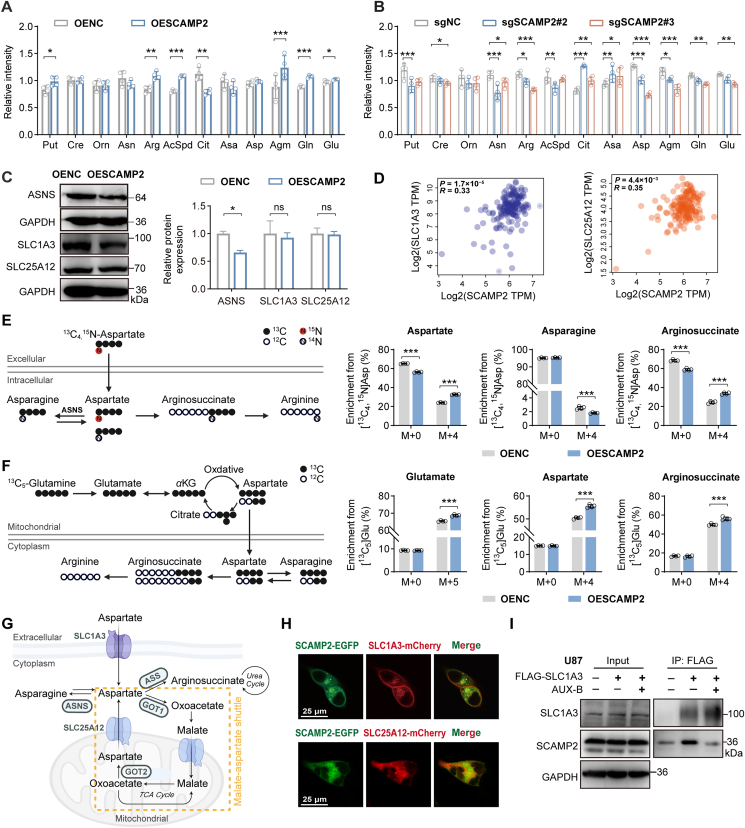
SCAMP2 reprogrammed aspartate metabolism mainly through aspartate transporters. (A) The relative intensities of metabolites in the aspartate–arginine metabolic pathway in OENC and OESCAMP2 groups in U87 cells (*n* = 4). Put, Putrescine; Cre, Creatine; Orn, l-Ornithine; Asn, l-Asparagine; Arg, l-Arginine; Ac-Spd, Ac-Spermidine; Cit, l-Citrulline; Asa, l-Argininosuccinate; Asp, l-Aspartate; Agm, Agmatine; Gln, l-Glutamine; Glu, l-Glutamate. (B) The relative intensities of metabolites in the aspartate-arginine metabolic pathway in sgNC, sgSCAMP2#2, and sgSCAMP2#3 groups in U87 cells are shown (*n* = 4). The metabolites indicated by the abbreviations are the same as in (A). (C) Western blot analysis of ASNS, SLC1A3, and SLC25A12 expression in OENC- and OESCAMP2-U87 cells (*n* ≥ 3). (D) Correlation analysis of mRNA expression of SLC1A3 and SLC25A12 with those of SCAMP2 in human GBM tissues in the TCGA database was performed. (E) The schematic diagram for the metabolism of [^13^C_4_,^15^N]-aspartate. White circles depict ^12^C, black solid circles depict ^13^C, N in white circles depicts ^14^N, and N in red circles depicts ^15^N. Mass isotopomer analysis of aspartate, asparagine, and arginosuccinate in cells overexpressing SCAMP2 or infected with an empty vector cultured under 10 mmol/L [^13^C,^15^N]-aspartate conditions was conducted (*n* = 4). (F) The schematic diagram for the metabolism of [^13^C_5_]-glutamine. White circles depict ^12^C and black solid circles depict ^13^C. Mass isotopomer analysis of glutamate, aspartate, and arginosuccinate in cells overexpressing SCAMP2 or infected with an empty vector cultured in 4 mmol/L [^13^C_5_]-glutamine conditions (*n* = 4). (G) Schematic depicting modulation of cytosolic aspartate levels. (H) HEK-293T cells were co-transfected transiently with SCAMP2-EGFP and SLC1A3-mCherry or SLC25A12-mCherry. The expressions of EGFP (left panel), mCherry (middle panel), and the overlay image (right panel) are shown for co-transfected cells (*n* = 3). (I) U87 cells were transfected with or without plasmid of FLAG-tagged SLC1A3, with or without treatment with AUX-B (1 μmol/L), then immunoblotting for indicated proteins after immunoprecipitation of FLAG from U87 cells, and levels of the co-immunoprecipitated SCAMP2 were detected with an anti-SCAMP2 antibody (*n* = 3). Data are given as mean ± SD, ∗*P* < 0.05, ∗∗*P* < 0.01 and ∗∗∗*P* < 0.001, analyzed by Student’s *t*-test or one-way ANOVA.

To confirm that aspartate metabolic reprogramming is mediated by SCAMP2 rather than being a non-specific consequence of AUX-B, we performed metabolomics analyses using GBM cell lines with SCAMP2 overexpression and knockdown and observed a consistent reduction in aspartate levels in knockdown groups (Fig. 5A and B). As expected, the protein expressions of ASNS exhibited a decreasing trend in the SCAMP2 overexpressing group and an increasing trend in the SCAMP2 knockdown group, while the expression levels of the aspartate transporters SLC1A3 and SLC25A12 remained unchanged (Fig. 5C, Supporting Information Fig. S15A and S15B). Interestingly, the TCGA database revealed that the mRNA levels of SCAMP2 are indeed positively correlated with SLC1A3 and SLC25A12 in GBM tissues (Fig. 5D). These results suggest that SCAMP2 regulates ASNS expression to influence cytoplasmic aspartate levels, but its effect on aspartate transport needs to be further confirmed.

To elucidate how SCAMP2 mediates aspartate metabolism, a metabolic flux analysis was performed on SCAMP2-overexpressing U87 cells. Aspartate is transported into the cytoplasm from the extracellular microenvironment *via* the plasma membrane transporter SLC1A3, which directly mediates its translocation^39^. When tracing [^13^C_4_,^15^N]-aspartate, the SCAMP2 overexpressed group exhibited a higher enrichment of aspartate [M+4] and arginosuccinate [M+4], alongside a lower enrichment of asparagine [M+4] (Fig. 5E). These observations indicate that SCAMP2 overexpression enhances the transport efficiency of plasma membrane transporters responsible for aspartate uptake. Concurrent changes in asparagine metabolic flux (Fig. 5E) and ASNS protein levels (Fig. 5C) further indicate that SCAMP2 overexpression reduces ASNS abundance and consequently decreases the rate of asparagine biosynthesis. Notably, the enrichment ratio of asparagine was relatively lower compared to other metabolites, suggesting that SCAMP2 overexpression exerts a modest but specific regulatory effect on asparagine metabolism. Additionally, aspartate is endogenously synthesized from oxaloacetate in mitochondria and subsequently transported to the cytoplasm *via* mitochondrial transporters. In the cytoplasm, aspartate serves as a critical precursor for purine and pyrimidine biosynthesis, an essential process supporting the abnormal proliferation of tumor cells^39^. To investigate this endogenous aspartate pathway, we traced the ^13^C-labeling ratios of metabolites involved in aspartate synthesis and metabolism (Fig. 5F). When [^13^C_5_]-glutamine was used as a tracer, carbon atoms were directly incorporated into glutamate, aspartate, and arginosuccinate. In the SCAMP2-overexpression group, the [M+5] enrichment ratio of glutamate and the [M+4] enrichment ratios of aspartate and arginosuccinate were all upregulated. This result indicates that SCAMP2 overexpression also enhances the transport efficiency of mitochondrial aspartate transporters. Collectively, these findings demonstrate that SCAMP2 overexpression simultaneously promotes aspartate transport efficiency *via* both plasma membrane and mitochondrial aspartate transporters. This dual enhancement leads to increased cytosolic aspartate accumulation while suppressing the metabolic conversion of aspartate to asparagine (Fig. 5G).

Since SCAMP2 is involved in membrane dynamics, secretion, and intracellular transport^15^^,^^40^, the co-localization and co-immunoprecipitation (Co-IP) were conducted to assess the interaction between SCAMP2 and transporters SLC1A3 and SLC25A12. The confocal imaging demonstrated that SCAMP2-GFP exhibited co-localization with SLC1A3-mCherry in the plasma membrane and intracellular vesicles and with SLC25A12-mCherry presumably in the mitochondrial membrane in HEK-293 T cells (Fig. 5H), and this observation is consistent in U87 and U251 cells (Fig. S15C and S15D). Further Co-IP assay confirmed that the exogenous (Fig. S15E) or endogenous (Fig. S15F) SCAMP2 interacted with SLC1A3, and AUX-B inhibited this coupling in U87 cells (Fig. 5I). To map the binding region between SCAMP2 and SLC1A3, we co-expressed full-length His-tagged SCAMP2 and a truncated version (the N-terminal 151-amino acid flexible peptide) with Flag-tagged SLC1A3. Co-IP assay demonstrated that SLC1A3 interacted with full-length SCAMP2 but not the truncation, indicating the binding region is not within the N-terminal 151-amino acid non-transmembrane domain (Fig. S15G). In fact, molecular dynamics simulations demonstrated that following the covalent modification of Lys130 by AUX-B, the 203–218 peptide segment in the transmembrane region underwent significant conformational changes, suggesting that this region may be involved in interactions with transporters (Fig. S15H and S15I)^40^. Therefore, SCAMP2-regulated aspartate metabolic reprogramming is primarily mediated through interactions with SLC1A3 and SLC25A12, which alter aspartate transport.

Collectively, our findings demonstrated that AUX-B, a conjugated polyene natural product, directly targets SCAMP2 in GBM cells and covalently binds to the non-transmembrane region of SCAMP2. This binding event thereby allosterically disrupts the interaction between SCAMP2 and two key aspartate transporters: SLC1A3 (localized to the plasma membrane) and SLC25A12 (localized to the mitochondrial membrane), consequently inhibiting aspartate transport. Notably, AUX-B targets SCAMP2 as well as promotes ASNS expression, thereby accelerating the metabolic consumption of aspartate for asparagine biosynthesis. Thus, AUX-B ultimately reduces cytosolic aspartate levels and subsequently inhibits GBM cell proliferation.

## 4 Discussion

GBM remains the most lethal primary brain tumor, with extremely limited treatment options. Temozolomide is still the only first-line postoperative chemotherapeutic agent used in clinical practice^41^. Drug development for GBM is hindered by four major bottlenecks: (1) the BBB restricts the penetration of most therapeutic agents; (2) genetic and metabolic heterogeneity limits the clinical applicability of targeted drugs; (3) the rapid development of drug resistance, and (4) the lack of specific, druggable molecular targets in GBM cells^42^. By contrast, natural products offer unique advantages for GBM therapy, including inherent BBB penetrability, multi-target regulatory effects, low systemic toxicity, and potential for synergistic combination strategies^22^.

In this study, we identified the natural product AUX-B, a polyenylpyrrole-type polyketide isolated from *Auxarthron* species, as a promising anti-GBM candidate. Our key finding include: (1) AUX-B efficiently across the BBB and accumulates in brain tissue; (2) it inhibits the proliferation and migration of multiple GBM cell lines *in vitro*; (3) it inhibits tumor growth in both U87-derived orthotopic and subcutaneous xenograft models (at a dose of 3.0 mg/kg); and (4) it exhibits no significant organ toxicity *in vivo*. Importantly, this is the first report of conjugated polyenes exerting anti-GBM activity, laying a foundation for the structural optimization of AUX-B and its translational research in GBM.

Although several reagents (*e*.*g*., temozolomide^43^, tyrosine kinase inhibitors, poly (ADP ribose) polymerase inhibitors, tubulin stabilizers, mTOR inhibitors, and glutathione transporter blockers^42^) have been explored for GBM treatment in clinical phases, durable disease control remains elusive, highlighting the urgent need for innovative targets and treatment strategies. Fungal secondary metabolites are a well-established source of clinical drugs (*e*.*g*., penicillin and lovastatin), and AUX-B represents a new member of this class with anti-GBM potential. Notably, while the U87 model is widely used in GBM research due to its well-defined genetics, stable tumorigenicity, and high reproducibility^44^, it cannot fully recapitulate clinical GBM features, such as interpatient heterogeneity, tumor microenvironment complexity, and variable therapeutic resistance^45^. Thus, future studies should validate AUX-B’s efficacy and mechanism using patient-derived xenografts (PDX) and genetically engineered mouse models (GEMM), which better mimic clinical scenarios.

SCAMP2 is known to regulate membrane trafficking, exocytosis, endocytosis, and cellular secretion^16^^,^^20^^,^^35^, but its role in GBM initiation and progression remained unclear before this work. Using ABPP strategies, we screened candidate proteins with molecular weights of 30–36 kDa and 40–55 kDa (prioritizing those with larger fold changes or significant *P*-values) for interaction with AUX-B (Fig. 2G), confirming SCAMP2 as a direct target of AUX-B in U87 cells. Rescue assays further supported this: SCAMP2 knockout or overexpression in U87 cells significantly interfered with AUX-B’s anti-tumor efficacy (Fig. S13D and S13E), indicating minimal off-target effects of AUX-B. However, considering the structural complexity of natural products and their potential for multi-target binding, we cannot exclude the existence of other weak interaction targets beyond SCAMP2.

SCAMP2 has previously been reported as a prognostic marker for acute myeloid leukemia^19^, but our study is the first to demonstrate that SCAMP2 is widely expressed in both human and mouse GBM tissues and functions as an oncogene, promoting GBM proliferation *in vitro* and *in vivo*. Previous work also showed that SCAMP2 regulates intracellular 5-HT and dopamine levels by modulating the serotonin transporter and the dopamine transporter^20^^,^^46^. Whether SCAMP2 regulates other intracellular metabolites, however, remains unknown.

Aspartate is an essential biosynthetic precursor for cancer cell proliferation^11^; it is also a rate-limiting metabolite for tumor growth under hypoxia^47^ or impaired electron transport chain activity (exogenous aspartate can restore proliferation in electron transport chain-deficient cells)^8^. Beyond proliferation, aspartate plays complex roles in tumor immunity: it drives lung metastasis and modulates the function of immune cells (like CD8^+^ T cells and macrophages) to regulate anti-tumor immune response^48^, ^49^, ^50^. Thus, targeting aspartate metabolism represents a potential strategy to inhibit GBM progression.

Using MSI, we found that aspartate levels are significantly higher in GBM tumors than in paracancerous brain tissues. To our knowledge, this is the first characterization of the aspartate metabolic landscape in GBM and its tumor microenvironment. Critically, AUX-B treatment significantly reduced these elevated aspartate levels (Fig. 4A, Table S5), a trend consistent with targeted metabolomic analysis of AUX-B-treated or SCAMP2-deficient cells (Fig. S14A and S14B, Fig. 5B). These data collectively confirmed that GBM relies on sustained high intracellular and intratumoral aspartate levels to support malignant proliferation, and that AUX-B exerts anti-GBM effects by targeting SCAMP2 to reprogram aspartate metabolism.

Cellular metabolic flux analysis further revealed that the mechanism by which SCAMP2 regulates aspartate metabolism: SCAMP2 overexpression enhanced aspartate transport across both the plasma membrane and mitochondrial membrane. This was evidenced by higher enrichment of [M+4]-aspartate (derived from [^13^C_4_,^15^N]-aspartate) and [M+4]-arginosuccinate (derived from [^13^C_5_]-glutamine) in SCAMP2-overexpressing cells (Fig. 5E and F). Notably, this mechanism differs from previously reported aspartate regulators (*e*.*g*., GOT1, SLC25A12), which directly modulate aspartate metabolism^8^^,^^10^, SCAMP2, by contrast, has no known transport activity; instead, it indirectly regulates cytosolic aspartate metabolism by modulating the function of downstream aspartate transporters, positioning SCAMP2 as a novel upstream regulator of aspartate metabolism in cancer.

Additionally, metabolic flux analysis showed reduced [M+4]-asparagine enrichment in SCAMP2 overexpressing cells (Fig. 5E), and Western blotting confirmed downregulated ASNS expression (Fig. 5C). This is particularly notable, as high ASNS expression drives asparagine synthesis to promote breast cancer brain metastasis and acute lymphoblastic leukemia progression^51^^,^^52^. Our findings suggest that when aspartate is scarce, promoting aspartate-to-asparagine conversion is not beneficial for GBM progression, providing new insight into the metabolic adaptability of GBM.

SCAMP2 is an integral membrane protein with four transmembrane helices, facilitating protein trafficking to the cell surface *via* recycling pathways^53^^,^^54^. It contains highly conserved regions, including the “E peptide” (between the second and third transmembrane helices), a domain critical for SCAMP2 function (*e*.*g*., mediating interactions with the serotonin transporter and the dopamine transporter to regulate neurotransmitter levels)^55^^,^^56^. Using Co-IP and fluorescence colocalization assay, we found that SCAMP2 directly interacts with SLC1A3 and SLC25A12 (Fig. 5H and I, Fig. S15E and S15F). To identify the SCAMP2 domain mediating these interactions, we tested a truncation mutant (N-terminal 151 amino acids); Co-IP results showed no interaction with SLC1A3 (Fig. S15G), ruling out this region. In fact, the 203–218 amino acid segment of the E peptide, enriched in basic and aromatic residues, has been shown to mediate SCAMP2’s interaction with SLC9A7^40^. These residues enable membrane binding and regulation of polyanionic phospholipid distribution^35^, suggesting the E peptide may also mediate SCAMP2’s interaction with SLC1A3/SLC25A12. Mechanistically, we found that AUX-B binds to Lys130 of SCAMP2 (a residue outside the E peptide) *via* MST assay (Fig. S12D), fluorescence labeling in-gel assay (Fig. 2L), and tandem MS/MS (Fig. 2K). Molecular dynamic simulation further showed that covalent modification of Lys130 by AUX-B induces significant conformation changes in the 203–218 E peptide segment (Fig. S15H and S15I). These results support a model where AUX-B binds to Lys130 and exerts long-range allosteric regulation of the E peptide, disrupting SCAMP2’s interaction with SLC1A3/SLC25A12 and thereby inhibiting aspartate transport. However, additional studies are needed to fully elucidate these allosteric regulatory mechanisms.

## 5 Conclusions

In summary, our study demonstrates AUX-B as a BBB-permeable, low-toxicity anti-GBM candidate; SCAMP2 as a novel GBM oncogene and upstream regulator of aspartate metabolism; and identifies a new mechanism by which SCAMP2 promotes GBM progression (*via* SLC1A3/SLC25A12-mediated aspartate transport) and is targeted by AUX-B. We also demonstrate that GBM relies on high aspartate levels for proliferation, and that restoring aspartate levels reverses AUX-B’s antitumor effects. These findings suggest that targeting SCAMP2 or aspartate transporters could be a viable strategy for GBM monotherapy or combination therapy with temozolomide, opening new avenues for GBM treatment.

## Author contributions

Changhui Shang: Data curation, Formal analysis, Writing-Original draft preparation, Writing-Reviewing and Editing. Wan Li: Investigation, Visualization, Validation. Qianlun Pu: Investigation, Formal Analysis, Data Curation. Bingyu Liu, Chen Zhang, Yiqing Tan, Yihui Yang: Visualization, Investigation. Dan Du: Supervision, Resources, Writing-Review & Editing, Project Administration. Jinhua Wang: Conceptualization, Supervision, Project Administration. Youcai Hu: Conceptualization, Methodology, Writing-Reviewing, and Editing. Funding Acquisition.

## Data availability

The data that support the findings of this study are available in the Supporting Information of this article: Supplementary Figures S1−S15; Sequences of RT-qPCR primers, Table S1; Key resources table, Table S2; NMR data distribution of compounds AUX-B and AUX-B-p, Table S3; Chemical proteomics data, Table S4; MSI data, Table S5; Targeted metabolomics data, Table S6; Proteomic data, Table S7.

## Conflicts of interest

The authors declare no competing interests.

## Appendix A Supporting information

The following are the Supporting Information to this article:
