# Supplementary material for: Targeting SCAMP2 by a natural product auxarconjugatin B for glioblastoma therapy via restoring aspartate metabolic flux
Source: Acta Pharm Sin B. 2026 Feb 27;16(5):3090–108. doi: 10.1016/j.apsb.2026.02.018 (PMC13198271; doi:10.1016/j.apsb.2026.02.018)
Supplement: Multimedia component 1 [file mmc1.pdf]

Supporting Information for

Original article

Targeting SCAMP2 by a natural product auxarconjugatin B for glioblastoma therapy *via* restoring aspartate metabolic flux

Changhui Shang[a,†], Wan Li [a,†], Qianlun Pu [b,†], Bingyu Liu[a], Chen Zhang[a], Yiqing Tan[a], Yihui Yang[a], Dan Du[b,*], Jinhua Wang[a,*], and Youcai Hu[a,*]

[a]*State Key Laboratory of Bioactive Substance and Function of Natural Medicines, Institute of Materia Medica, Chinese Academy of Medical Sciences & Peking Union Medical College, Beijing 100050, China*

[b]*Advanced Mass Spectrometry Center, Research Core Facility, Frontiers Science Center for Disease-related Molecular Network, West China Hospital, Sichuan University, Chengdu 610041, China*

Received 24 April 2025; received in revised form 4 October 2025; accepted 5 November 2025

*Corresponding authors.

E-mail addresses: huyoucai@imm.ac.cn (Youcai Hu), wjh@imm.ac.cn (Jinhua Wang), dudan1520@163.com (Dan Du).

[†]These authors made equal contributions to this work.

This file contains the following Supporting Information for Figures, Materials, and Tables:

1. Supplementary Figures

Page 3: Figure S1. The ^{1}H NMR (500Hz) spectrum of AUX-B in DMSO-d_6.

Page 3: Figure S2. The ^{13}C NMR (125Hz) spectrum of AUX-B in DMSO-d_6.

Page 4: Figure S3. The HR-ESI-MS of AUX-B.

Page 4: Figure S4. The ^{1}H NMR (500Hz) spectrum of AUX-B-p in DMSO-d_6.

Page 5: Figure S5. The ^{13}C NMR (125Hz) spectrum of AUX-B-p in DMSO-d_6.

Page 5: Figure S6. ^{1}H–^{1}H COSY (500Hz) spectrum of AUX-B-p in DMSO-d_6.

Page 6: Figure S7. HSQC (500Hz) spectrum of AUX-B-p in DMSO-d_6.

Page 6: Figure S8. HMBC (500Hz) spectrum of AUX-B-p in DMSO-d_6.

Page 7: Figure S9. The HR-ESI-MS of AUX-B-p.

Page 8: Figure S10. AUX-B inhibited DNA synthesis, migration, and invasion of GBM cells.

Page 9: Figure S11. AUX-B exhibits anti-glioblastoma activity *in vivo*.

Page 11: Figure S12. Identification of SCAMP2 as a direct target of AUX-B.

Page 12: Figure S13. SCAMP2 is highly expressed in GBM tumor tissues.

Page 13: Figure S14. Inhibition of SCAMP2 by AUX-B induced aspartate metabolic dysfunction.

Page 15: Figure S15. SCAMP2 reprogrammed aspartate metabolism mainly through the aspartate transporter.

2. Supplementary Tables

Page 17: Table S1. Sequences of RT-qPCR primers.

Page 19: Table S2. Key resources table.

Page 18: Table S3. ^{1}H NMR and ^{13}C NMR spectroscopic data (δ) for compounds AUX-B and AUX-B-p (δ in ppm).

Table S4. Chemical proteomics data, see excel file.

Table S5. MSI data, see excel file.

Table S6. Proteomic data, see excel file.

Table S7. Targeted metabolomics data are in other files, see excel file.

.

Supplementary Figures

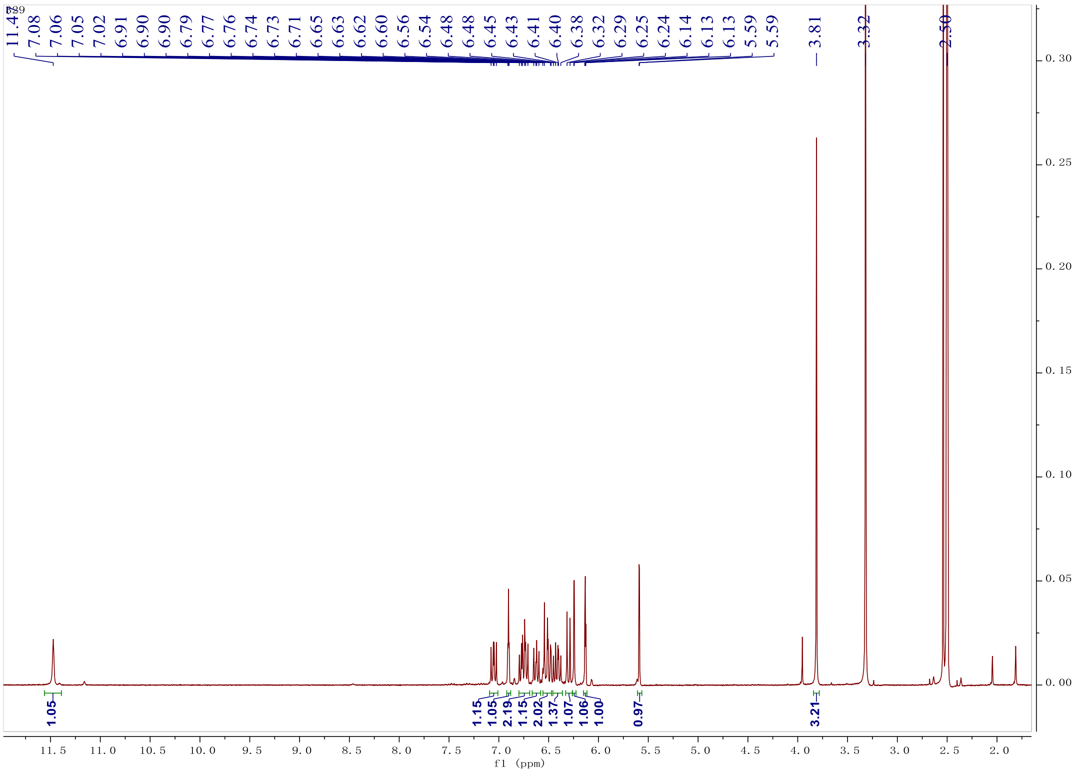

Figure S1. The ^{1}H NMR (500 Hz) spectrum of AUX-B in DMSO-d_6

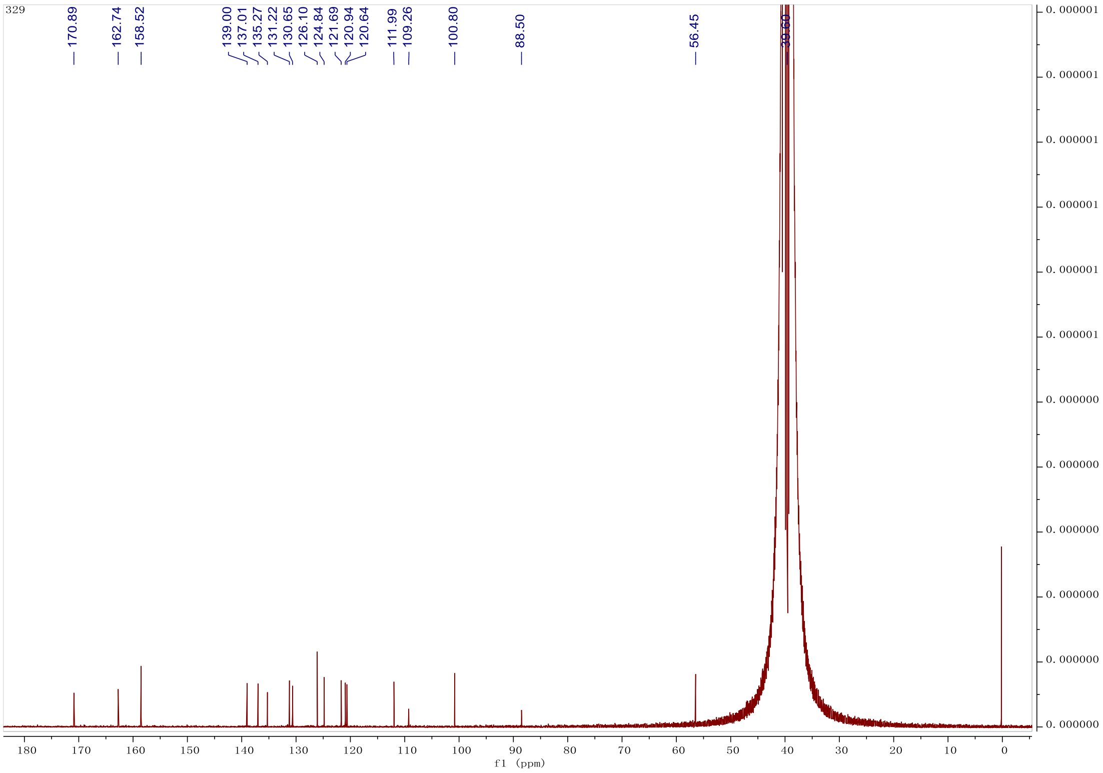

Figure S2. The ^{13}C NMR (125 Hz) spectrum of AUX-B in DMSO-d_6

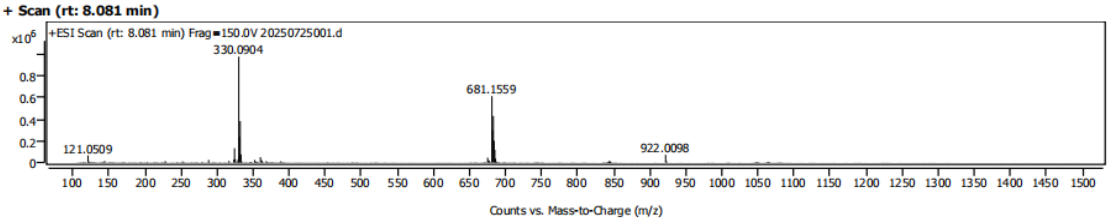

Spectrum Peaks

m/z	Z	Abund	Abund %	m/z (Calc)	Diff (ppm)	Ion Species	Formula	Ion Type
330.0904	1	979307	100.00	330.0891	3.73	(M+H)+	C18 H16 Cl N O3	
331.0927	1	236690	24.17	331.0924	0.88	(M+H)+	C18 H16 Cl N O3	
332.0879	1	382771	39.09	332.0869	3.19	(M+H)+	C18 H16 Cl N O3	
333.0901	1	74489	7.61	333.0898	1.07	(M+H)+	C18 H16 Cl N O3	
121.0509		65288	6.67					
324.1595		132537	13.53					
330.2501		62587	6.39					
360.0995		49363	5.04					
681.1559	1	613940	62.69					
682.1575	1	298105	30.44					
683.1555	1	429636	43.87					
684.1546	1	201121	20.54					
685.1509	1	116871	11.93					
922.0098		71435	7.29					

Spectrum Identification Table

Best	ID Source	Name	Formula	Species	m/z	Diff (ppm)	CAS	Score	Score (Lib)	Score (DB)	Score (MFG)	Lib/DB
No	MFG		C18 H16 Cl N O3	(M+H)+	330.0904	3.07		99.46			99.46	

Figure S3. The HR-ESI-MS of AUX-B.

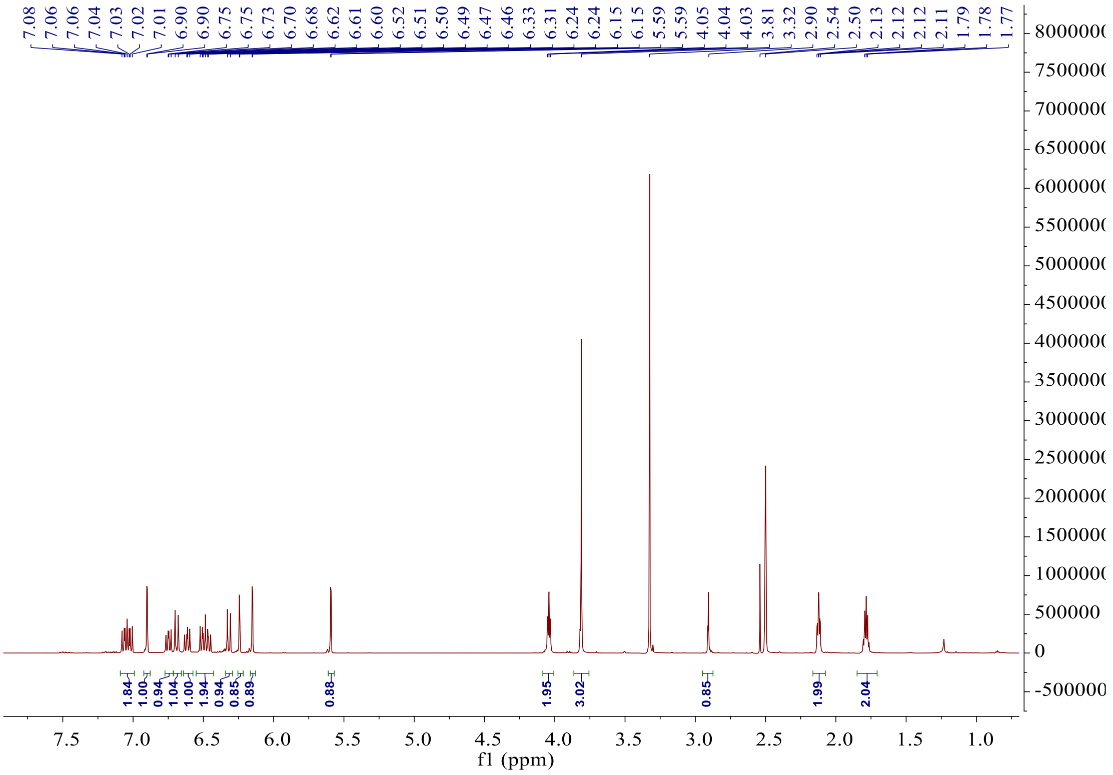

Figure S4. The ^{1}H NMR (500 Hz) spectrum of AUX-B-p in DMSO-d_6

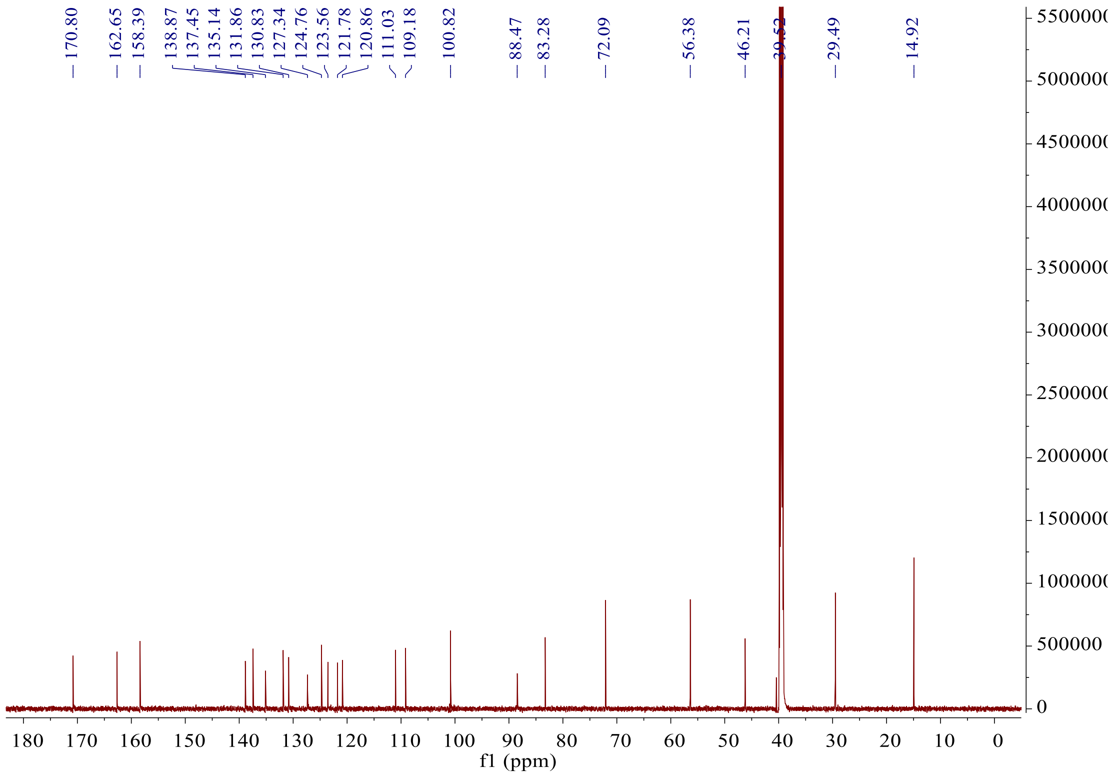

Figure S5. The ^{13}C NMR (125 Hz) spectrum of AUX-B-p in DMSO-d_6

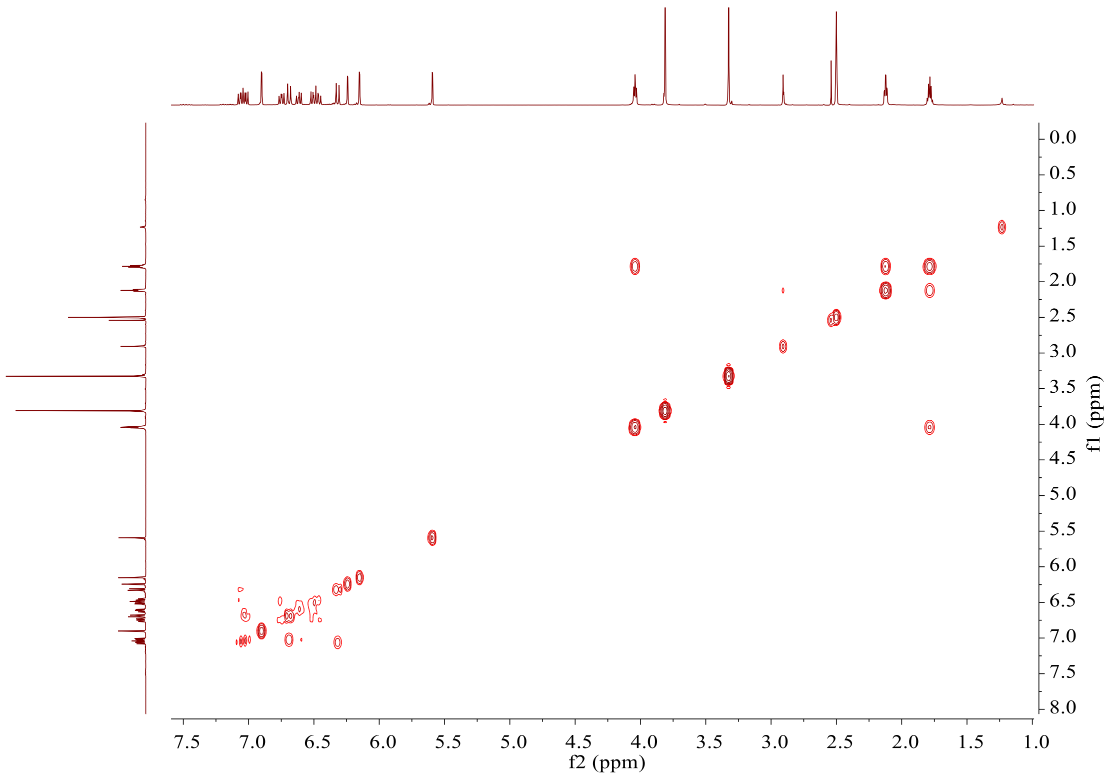

Figure S6. ^{1}H–^{1}H COSY (500 Hz) spectrum of AUX-B-p in DMSO-d_6

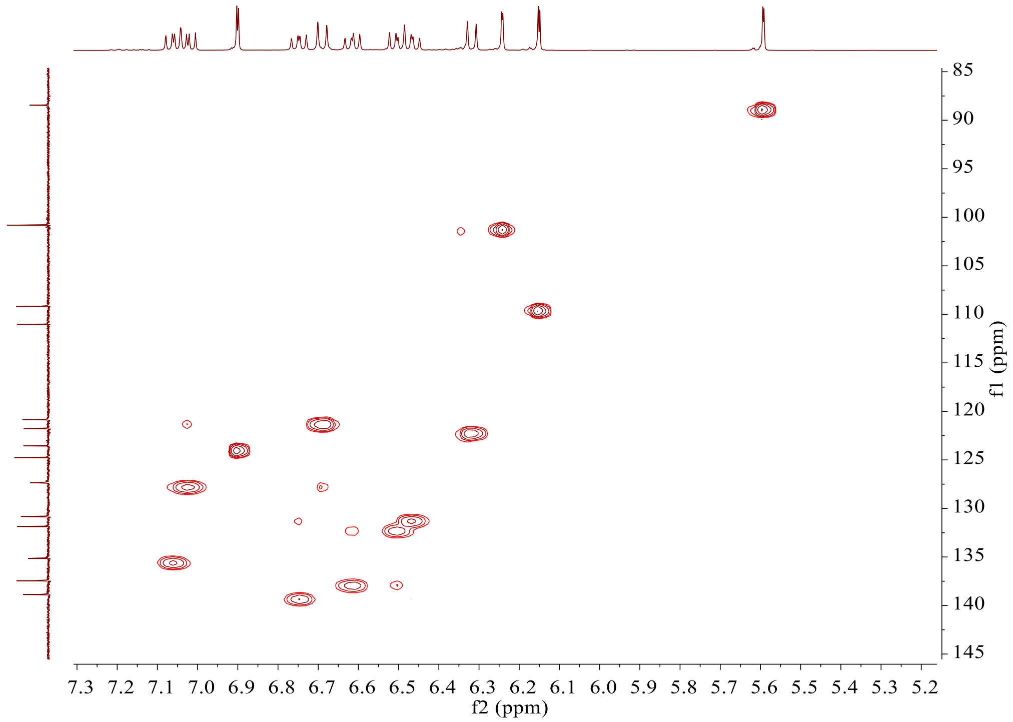

Figure S7. HSQC (500 Hz) spectrum of AUX-B-p in DMSO-d_6

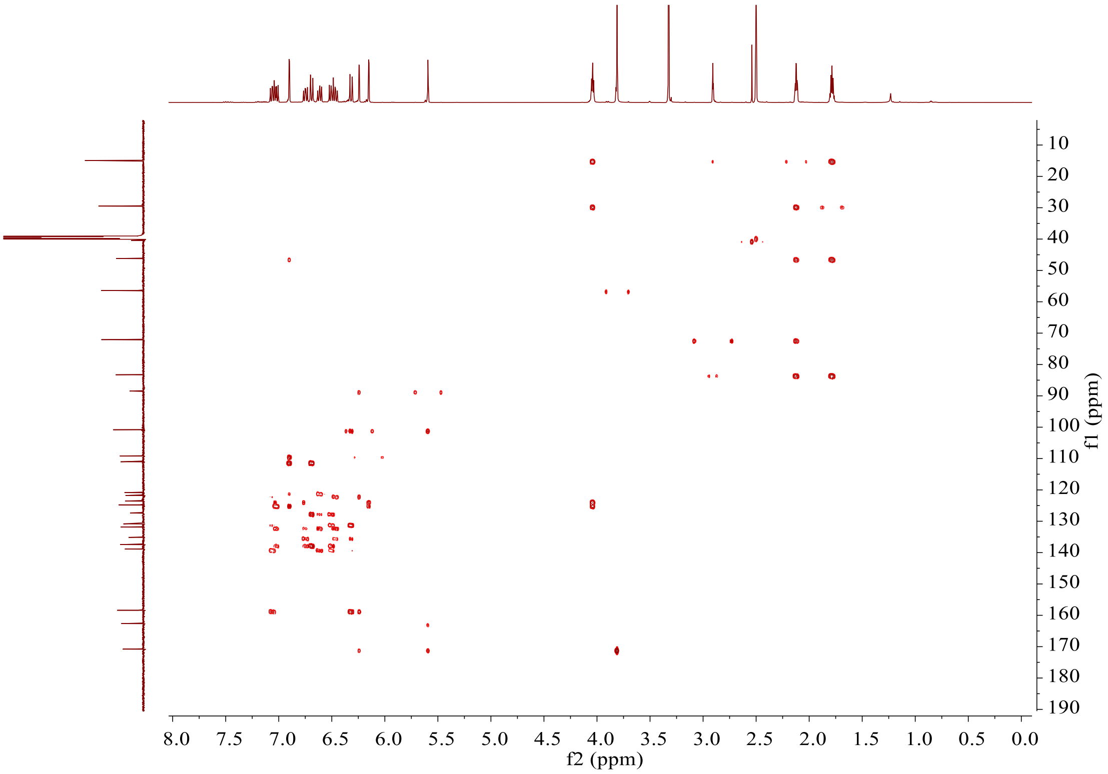

Figure S8. HMBC (500 Hz) spectrum of AUX-B-p in DMSO-d_6

+ Scan (rt: 8.823 min) **C25 H24 Cl N3 O3**

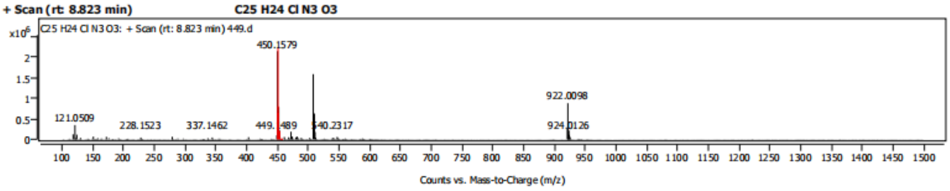

Spectrum Peaks

m/z	Z	Abund	Abund %	m/z (Calc)	Diff (ppm)	Ion Species	Formula	Ion Type
450.1579	1	2134359	100.00	450.1579	-0.07	(M+H)+	C25 H24 Cl N3 O3	
451.1603	1	674656	31.61	451.1610	-1.61	(M+H)+	C25 H24 Cl N3 O3	
452.1562	1	780182	36.55	452.1561	0.38	(M+H)+	C25 H24 Cl N3 O3	
453.1580	1	214883	10.07	453.1586	-1.31	(M+H)+	C25 H24 Cl N3 O3	
454.1680	1	51064	2.39	454.1612	14.98			
118.0860		120691	5.65					
121.0509		344430	16.14					
122.0952		22522	1.06					
124.0867		112037	5.25					
130.1588		33390	1.56					
151.0321		64872	3.04					
158.1530		26031	1.22					
172.1693		59154	2.77					
176.1388		26820	1.26					
228.1523		42118	1.97					
279.1588		59814	2.80					
337.1462		23943	1.12					
344.2248		43467	2.04					
403.2325		50579	2.37					
449.1489		96429	4.52					
461.2853		54929	2.57					
468.1673		48217	2.26					
472.1393	1	183709	8.61					
473.1425	1	51798	2.43					
474.1379	1	63687	2.98					
480.1706		47742	2.24					
482.1795		63910	2.99					
488.1130		25497	1.19					
502.1982		29525	1.38					
508.2109	1	1577890	73.93					
509.2136	1	538711	25.24					
510.2088	1	624326	29.25					
511.2108	1	176150	8.25					
512.2162	1	37831	1.77					
540.2317		31361	1.47					
542.2302		21456	1.01					
547.2204		55408	2.60					
921.2881	1	254674	11.93					
922.0098	1	874866	40.99					
922.2865	1	179698	8.42					
923.0112	1	192369	9.01					
923.2856	1	221458	10.38					
924.0126	1	25984	1.22					
924.2882	1	107838	5.05					
925.2871	1	56659	2.65					
926.2872	1	21954	1.03					

Spectrum Identification Table

Best	ID Source	Name	Formula	Species	m/z	Diff (ppm)	CAS	Score	Score (Lib)	Score (DB)	Score (MFG)	Lib/DB
Yes	MFG		C25 H24 Cl N3 O3	(M+H)+	450.1579	0.02		98.46			98.46	

MassHunter Qual 10.0
(End of Report)

Figure S9. The HR-ESI-MS of AUX-B-p.

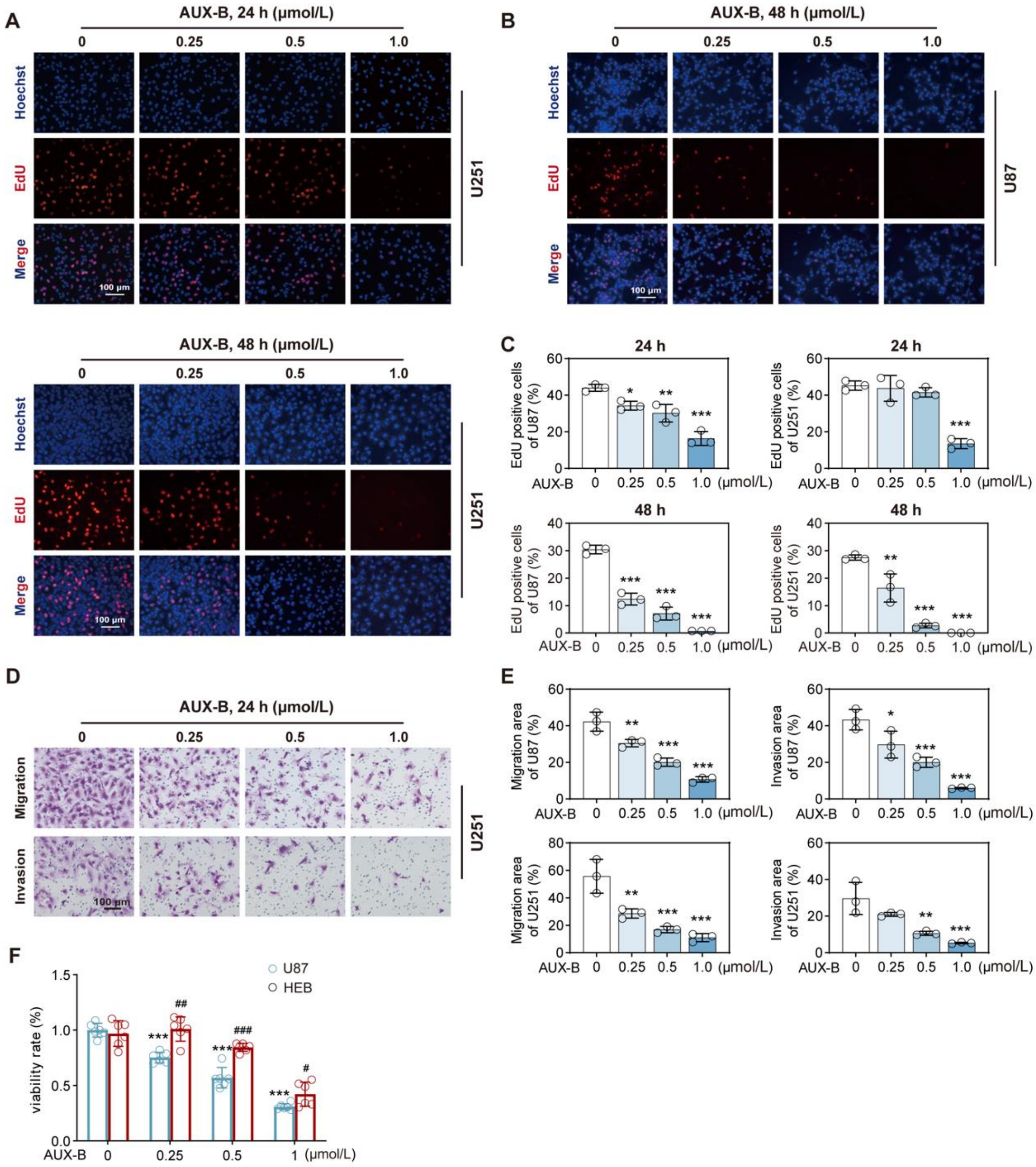

Figure S10. AUX-B inhibited DNA synthesis, migration, and invasion of GBM cells. (A) The EdU assay of U251 cells after treatment with 0, 0.25, 0.5, and 1.0 μmol/L AUX-B for 24 and 48 h. **(B)** The EdU assay of U87 cells after treatment with 0, 0.25, 0.5, and 1.0 μmol/L AUX-B for 48 h. **(C)** Quantitative analysis of the EdU assay for U87 and U251 cells. **(D)** The migration and invasion of U251-MG cells treated with AUX-B were assessed by transwell assay. **(E)** Quantitative analysis of the transwell assay for U87 and U251 cells. (**F**) U87 and HEB cells were treated with AUX-B for 24 h, respectively, and then the viability was determined using the CCK-8 assay. Data are presented as mean ± SD, **P* < 0.05, ***P* < 0.01, and ****P* < 0.001, *vs* the control group, and #*P* < 0.05, ##*P* < 0.01, and ###*P* < 0.001 *vs* the U87 group, as determined by Student's *t*-test or one-way ANOVA.

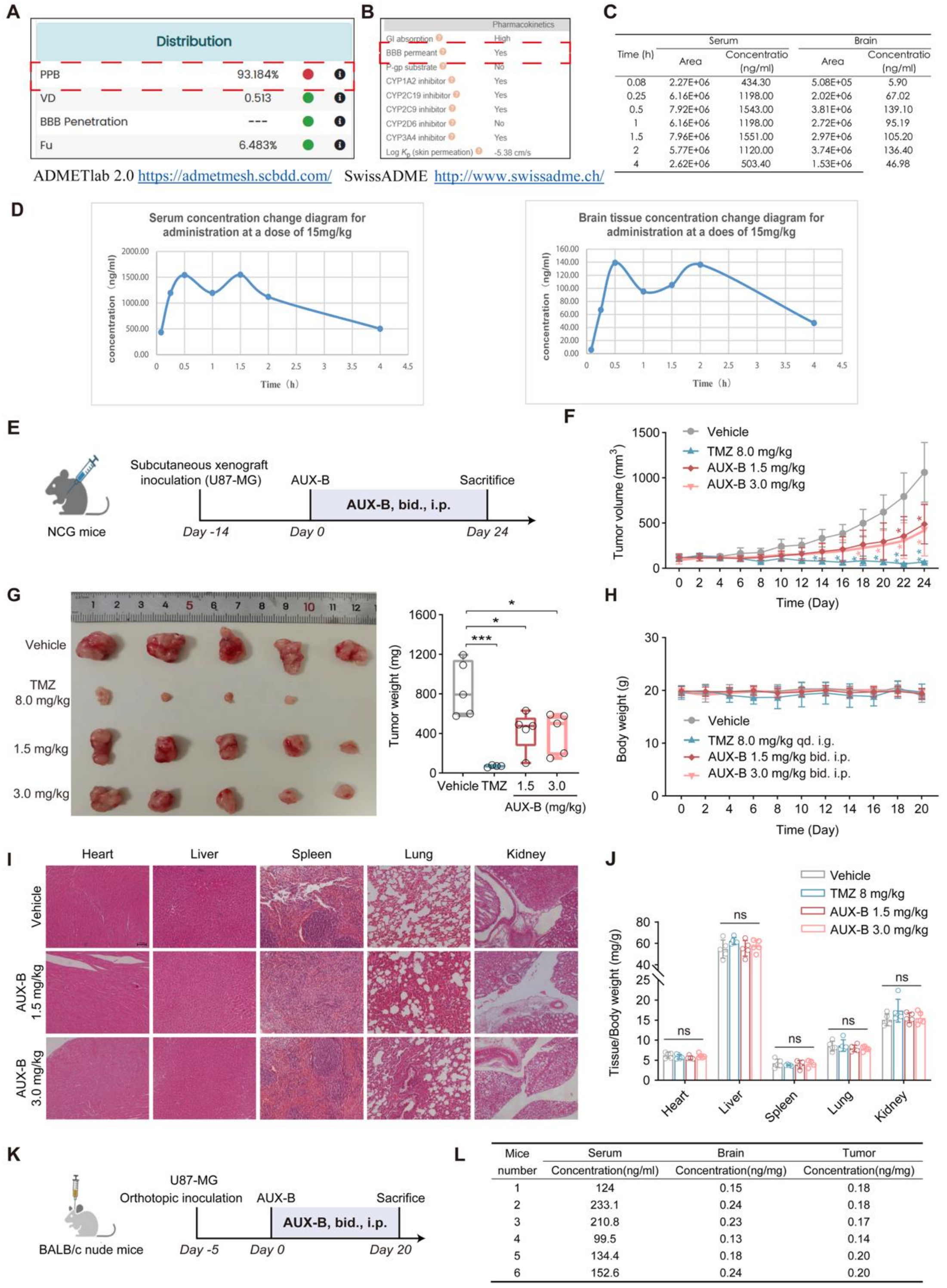

Figure S11. AUX-B exhibits anti-glioblastoma activity *in vivo*. (A, B) Prediction of the ability of AUX-B to penetrate the blood–brain barrier (BBB) using ADME websites. **(C)** Concentrations of AUX-B in serum and brain tissue at different time points after intraperitoneal administration of 15 mg/kg AUX-B to mice. **(D)** Serum (left panel) and brain tissue (right panel) concentrations change diagram after intraperitoneal administration of 15 mg/kg AUX-B to mice. **(E)** Schematic outlines of the construction of the U87-derived subcutaneous tumor model in NCG mice. The mice were treated with either 8 mg/kg TMZ (qd, i.g.), 1.5 mg/kg AUX-B (bid, i.p.), 3.0 mg/kg AUX-B (bid,

i.p.), or solvent (bid, i.p.) as the vehicle group. **(F)** Growth curve of U87-derived subcutaneous tumor model during the administration period (*n* = 5). **(G)** The image (left panel) and the tumor weight (right panel) of U87-derived subcutaneous tumor model (*n* = 5). **(H)** Body weight changes in vehicle and AUX-B treated groups in U87-derived orthotopic tumor models (*n* = 5). **(I)** Hematoxylin–eosin (H&E) staining of heart, liver, spleen, lung, and kidney from the U87-derived orthotopic tumor model (*n* = 3). **(J)** The visceral-body ratio of the U87-derived orthotopic tumor model (*n* = 5). **(K)** Schematic outlines of the construction of U87-derived orthotopic tumor model in BALB/c nude mice. The mice were treated with either AUX-B (3.0 mg/kg twice daily i.p. injection), or solvent (twice daily, i.p. injection) as the vehicle group. Thirty minutes after the final drug administration, the animals were euthanized. Subsequently, serum, brain and tumor samples were collected, and the content of AUX-B in these samples was detected *via* LC–MS. **(L)** Determination of AUX-B levels in serum, brain, and tumor samples from BALB/c mice at therapeutic dose (3 mg/kg) *via* LC–MS. Data are presented as mean ± SD, $^{*}P < 0.05$, $^{**}P < 0.01$ and $^{***}P < 0.001$, as determined by one-way ANOVA or two-way ANOVA.

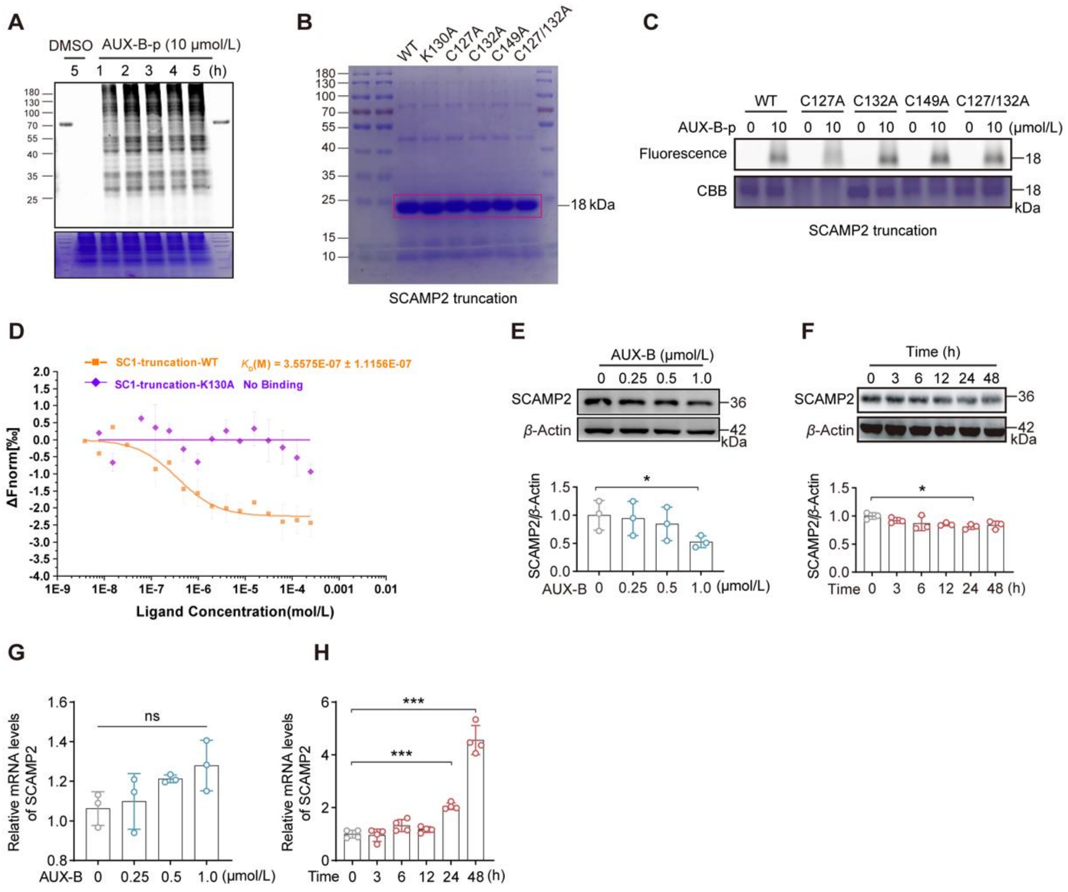

Figure S12. Identification of SCAMP2 as a direct target of AUX-B. (A) *In situ* time-dependent ABPP labeling of U87 cells with AUX-B-p probe. **(B)** Analysis of the recombinant SCAMP2 mutant truncation by SDS-PAGE. **(C)** In-gel fluorescence labeling of recombinant SCAMP2 truncations and various mutants with AUX-B-p. (**D**) MST assay of AUX-B binding to recombinant SCAMP2 truncation (1–151) wildtype and K130A mutant version. Western blot analysis and quantitation of SCAMP2 expression in U87 cells treated with indicated concentrations of AUX-B for 48 h **(E)** and with AUX-B at a concentration of 2.0 μmol/L for different time points **(F)**. Quantitative PCR analysis of SCAMP2 in U87 cells after treatment with indicated concentrations of AUX-B for 48 h **(G)** and with AUX-B at a concentration of 2.0 μmol/L for indicated time points **(H)**. Data are presented as mean ± SD, **P* < 0.05 and *** *P* < 0.001 determined by Student's *t*-test or one-way ANOVA.

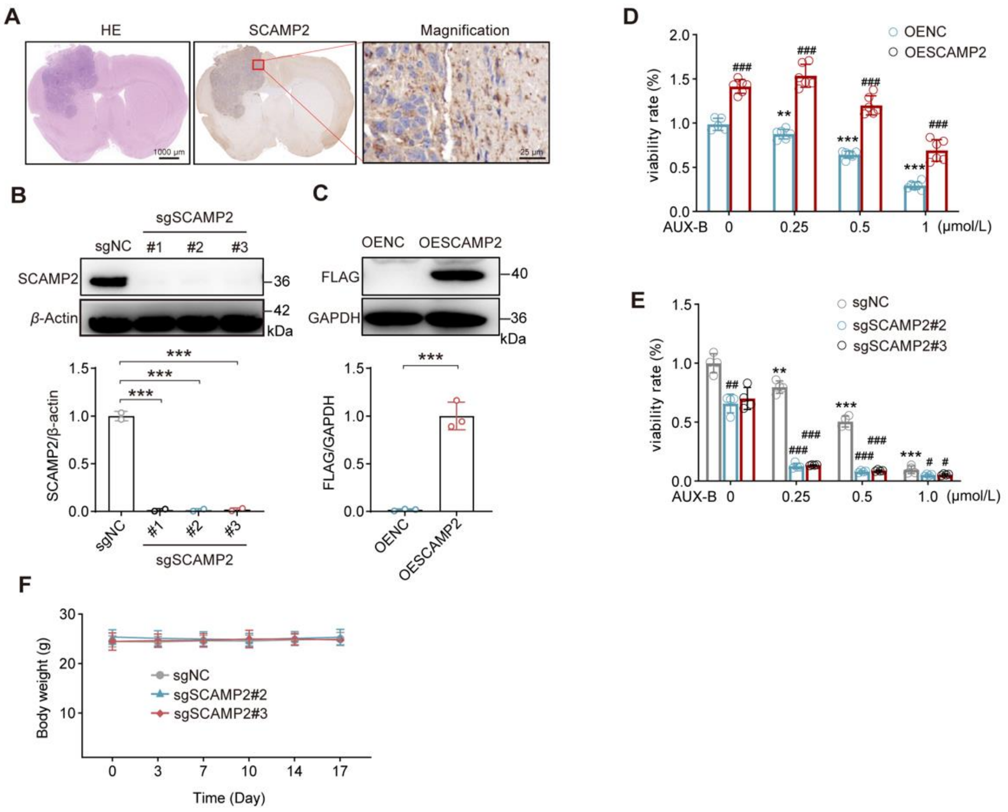

Figure S13. SCAMP2 is highly expressed in GBM tumor tissues. (A) The pathology and expression of SCAMP2 were assessed by H&E and immunohistochemical staining in GL261-derived orthotopic tumor model (*n* = 4). The left picture represents hematoxylin–eosin (H&E) staining and the middle picture represents IHC staining (scale bar: 100 μm). The right pictures are the enlarged representations of the boxed regions of the middle pictures (scale bar: 25 μm). **(B)** Western blot analysis and quantitation of SCAMP2 in negative control (NC) and SCAMP2 knockout groups in U87 cells. **(C)** Western blot analysis and quantitation of Flag expression in NC and SCAMP2 overexpression groups in U87 cells. The SCAMP2 overexpression in U87 cells was generated by lentiviral transduction with a plasmid containing a FLAG tag fused to SCAMP2, so the Western blot of FLAG indicates the presence of SCAMP2. U87 cells with overexpression of SCAMP2 (**D**) or knockdown of SCAMP2 (**E**) were treated with AUX-B for 24 h, and then the viability was determined using the CCK-8 assay. **(F)** Changes in body weight were monitored in sgNC and SCAMP2-knockout U87-derived subcutaneous xenograft tumor mouse models (*n* = 6). Data are presented as mean ± SD, $^{*}P < 0.05$, $^{**}P < 0.01$, and $^{***}P < 0.001$ *vs* the control group, and $^{\#}P < 0.05$, $^{\#\#}P < 0.01$, and $^{\#\#\#}P < 0.001$ *vs* the sgNC or OENC group determined by Student's *t*-test or one-way ANOVA.

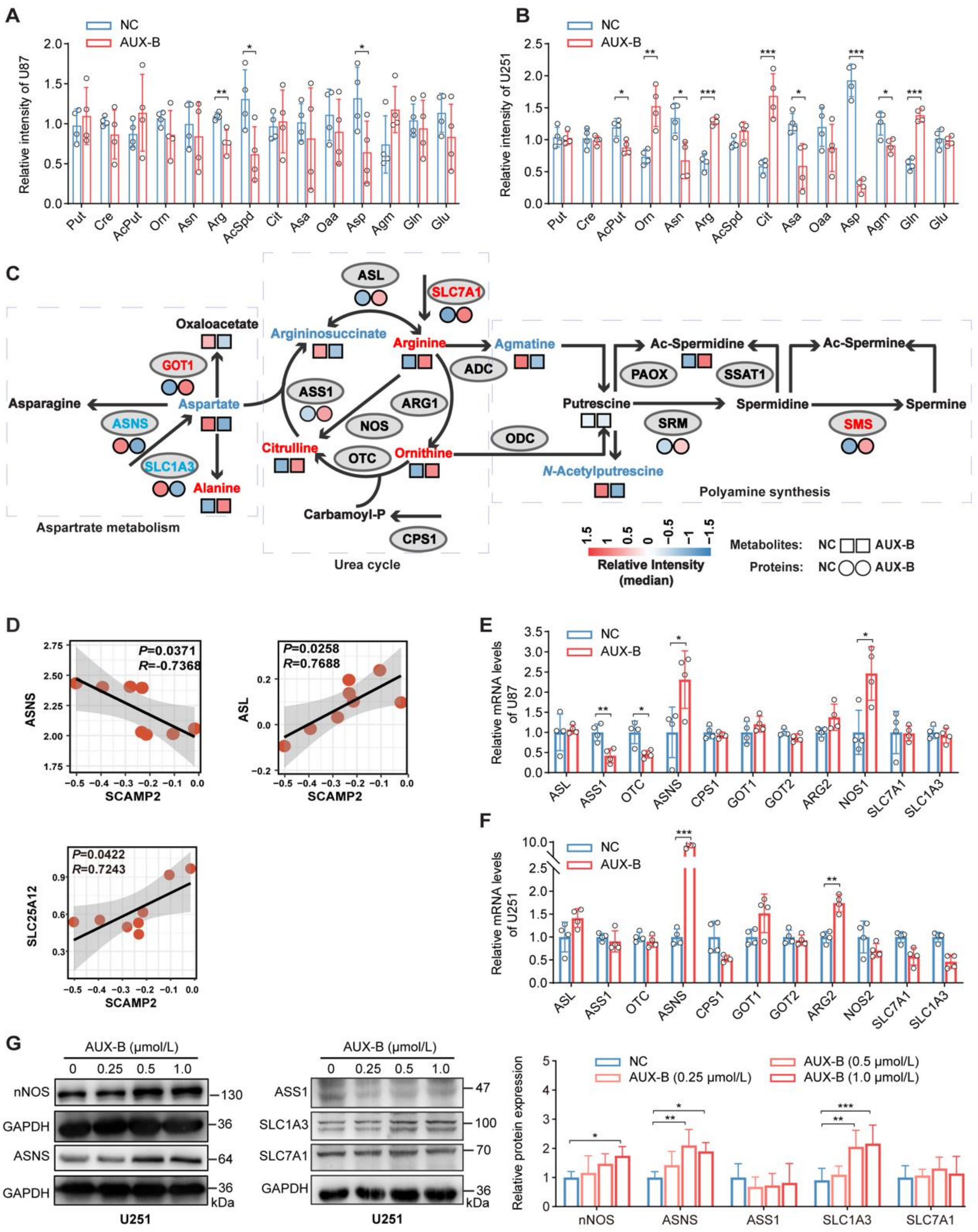

Figure S14. Inhibition of SCAMP2 by AUX-B induced aspartate metabolic dysfunction. Changes of metabolite levels related to arginine and aspartate metabolism in U87 **(A)** and U251 **(B)** cells, with or without 24-h treatment of AUX-B (1 μmol/L) (n = 4). Put, Putrescine; Cre, Creatine; Ac-Put, Ac- Putrescine; Orn, L-Ornithine; Asn, L-Asparagine; Arg, L-Arginine; Ac-Spd, Ac-Spermidine; Cit, L-Citrulline; Asa, L-Argininosuccinate; Oaa, Oxalacetate; Asp, L-Aspartate; Agm, Agmatine; Gln, L-Glutamine; Glu, L-Glutamate. **(C)** A schematic diagram illustrates the metabolism of aspartate–arginine. The circles beneath the enzymes and boxes beneath the metabolites represent the alterations in protein and metabolite levels in U251 cells treated with (right) or without (left) 1 μmol/L AUX-B, respectively. Color coding reflects the level of log_2-fold

change as indicated (n = 4). nNOS, neuronal nitric oxide synthase; ASNS, asparagine synthetase; ASS1, argininosuccinate synthetase 1; SLC1A3, solute carrier family 1 member 3; SLC7A1: solute carrier family 7 member 1. **(D)** The correlation analysis of ASNS, ASL, and SLC25A12 with SCAMP2 in proteomics after AUX-B treatment in U251 cells. ASL, argininosuccinate lyase; SLC25A12, solute carrier family 25 member 12. Quantitative PCR analysis of enzymes related to aspartate and arginine metabolism in U87 **(E)** and U251 **(F)** cells after treatment with AUX-B (1 μmol/L) for 24 h (n = 4). **(G)** Western blot analysis and quantitation of enzymes and transporters associated with aspartate and arginine metabolism in U251 cells treated with various doses of AUX-B (n > 3). Data are presented as mean ± SD, *P < 0.05, **P < 0.01, and ***P < 0.001 determined by Student's t-test or one-way ANOVA.

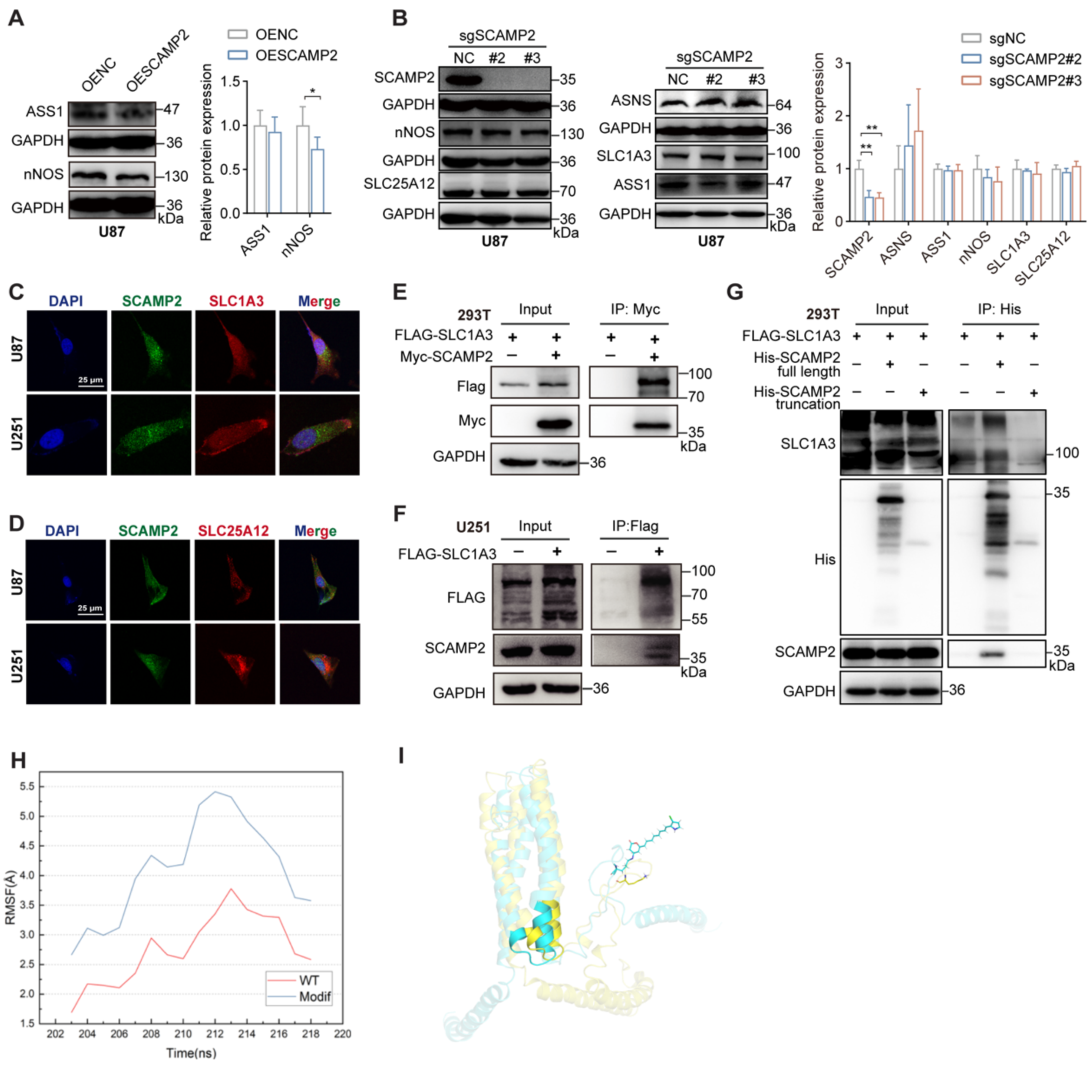

Figure S15. SCAMP2 reprogrammed aspartate metabolism mainly through the aspartate transporter. (A) Western blot analysis of ASS1 and nNOS in OENC and OESCAMP2 U87 cells ($n > 3$). **(B)** Western blot analysis and quantitation of enzymes and transporters related to aspartate metabolism in sgNC and sgSCAMP2 U87 cells ($n = 3$). Immunofluorescence co-localization of SCAMP2 with SLC1A3 **(C)** or SLC25A12 **(D)** in U87 and U251 cells, respectively. **(E)** The interaction of SCAMP2 and SLC1A3 was evaluated by Co-IP assays in 293T cells. 293T cells were transfected with FlAG-tagged SLC1A3 and Myc-tagged SCAMP2, followed by immunoprecipitation using Myc beads, and levels of the co-immunoprecipitated FLAG-tagged SLC1A3 were detected with an anti-FLAG antibody. **(F)** The interaction of SCAMP2 and SLC1A3 was evaluated by Co-IP assays in U251 cells. U251 cells were transfected with FLAG-tagged SLC1A3 for 24 h, and immunoblotting was performed for the indicated proteins after immunoprecipitation with FLAG beads from U251 cells. **(G)** The interaction of SCAMP2 full length/truncation and SLC1A3 was evaluated by Co-IP assays in 293T cells. 293T cells were transfected with FLAG-tagged SLC1A3 and His-tagged SCAMP2 full length/truncation, followed by immunoprecipitation with His beads, and the levels of co-immunoprecipitated Flag-SLC1A3 were detected using an anti-SLC1A3 antibody. **(H)** Root Mean Square Fluctuation (RMSF) is used

to evaluate the fluctuation amplitude of each residue during the simulation, thereby revealing which domains of SCAMP2 become more flexible or exhibit increased stability following covalent modification by AUX-B. **(I)** Overlaid snapshots of the 200 ns trajectory for the two states of the SCAMP2 protein in molecular dynamics simulation: whether its Lys130 site is covalently modified by AUX-B (modified *vs*. unmodified). The yellow cartoon represents the conformation of unmodified SCAMP2, while cyan represents the conformation of modified SCAMP2. The non-transparent region indicates the domain spanning residues 203–218. Data are presented as mean ± SD, $^*P < 0.05$ determined by one-way ANOVA.

Table S1. Sequences of RT-qPCR primers.

Gene	Primer
SCAMP2	5′–3′ ATGCAGCGACAACAGTTCCT<br>3′–5′ GCTGGGTTGGTTCCACTGA
β-Actin	5′–3′ CATGTACGTTGCTATCCAGGC<br>3′–5′ CTCCTTAATGTCACGCACGAT
SLC1A3	5′–3′ AGCAGGGAGTCCGTAAACG<br>3′–5′ AGCATTCCGAAACAGGTAACTTT
SLC7A1	5′–3′ GTCCTGCTCAACATTGGGCA<br>3′–5′ CAGGGCCTGCATTCTCACG
ASNS	5′–3′ GGAAGACAGCCCCGATTTACT<br>3′–5′ AGCACGAACTGTTGTAATGTCA
ASL	5′–3′ GCCGAGATGGACCAGATACTC<br>3′–5′ CTGCCGTTGCACCAATGAG
CPS1	5′–3′ AATGAGGTGGGCTTAAAGCAAG<br>3′–5′ AGTTCCACTCCACAGTTCAGA
NOS2 (*iNOS*)	5′–3′ TTCAGTATCACAACCTCAGCAAG<br>3′–5′ TGGACCTGCAAGTTAAAATCCC
ARG2	5′–3′ CGCGAGTGCATTCCATCCT<br>3′–5′ TCCAAAGTCTTTTAGGTGGCAG
OTC	5′–3′ CGGCCCGTGTATTGTCTAGC<br>3′–5′ TAGCCAGGGTGTCCAAATCTG
ASS1	5′–3′ TCCGTGGTTCTGGCCTACA<br>3′–5′ GGCTTCCTCGAAGTCTTCCTT
GOT1	5′–3′ ATGGCACCTCCGTCAGTCT<br>3′–5′ AGTCATCCGTGCGATATGCTC
GOT2	5′–3′ AAGAGGGACACCAATAGCAAAAA<br>3′–5′ GCAGAACGTAAGGCTTTCCAT
NOS1 (*nNOS*)	5′–3′ TTCCCTCTCGCCAAAGAGTTT<br>3′–5′ AAGTGCTAGTGGTGTCGATCT

Table S2. Key resources table.

REAGENT or RESOUCE	SOURCE	IDENTIFIER
**Antibodies**		
ASNS antibody	Cell Signaling Technology	Cat# 92479T
nNOS antibody	Abcam	Cat# ab307562
ASS1 antibody	Abcam	Cat# ab170952
SCAMP2 antibody	Thermo Fisher Scientific	Cat# MA5-26296
SCAMP2 antibody	Abcam	Cat# 1078861-1
SCAMP2 antibody	ImmunoWay	Cat# YN7405
SLC7A1 antibody	ABclonal Technology	Cat# A25422
SLC1A3 antibody	Proteintech	Cat# 20785-1-AP
SLC25A12 antibody	ABcolonal Technology	Cat# A21129
His-Tag antibody	Proteintech	Cat# 66005-1-Ig
FLAG antibody	Beyotime	Cat# AG8050
MYC-Tag antibody	Proteintech	Cat# 60003-2-Ig
Beta Actin antibody	Proteintech	Cat# 66009-1-Ig
GAPDH antibody	Proteintech	Cat# 60004-1-Ig
Anti-rabbit IgG, HRP-linked antibody	Cell Signaling Technology	Cat# 7074P2
Anti-mouse IgG, HRP-linked antibody	Cell Signaling Technology	Cat# 7076P2
Alexa Fluor ™ 488 goat anti-rabbit secondary antibody,	Thermo Fisher Scientific	Cat# A11008
Alexa Fluor ™ 488 goat anti-mouse secondary antibody	Thermo Fisher Scientific	Cat# A11001
Alexa Fluor ™ 532 goat anti-mouse secondary antibody	Thermo Fisher Scientific	Cat# A11002
Alexa Fluor ™ 532 goat anti-Rabbit secondary antibody	Thermo Fisher Scientific	Cat# A11005
**Chemicals, peptides, and recombinant proteins**		
DMEM, High Glucose	Transgene	Cat# Fl101-01
Fetal Bovine Serum	Pricella	Cat# 164210-50
Bovine serum albumin V	Solarbio LIFE SCIENCE	Cat# A8026
Trypsin (2.5%+EDTA)	Transgen biotech	Cat# FG301-01
1% penicillin and streptomycin	Yeasen	Cat# FG101-01
RIPA lysis buffer	Applygen	Cat# C1053-100
IP lysis buffer	Applygen	Cat# C1054
Dual Color SDS-PAGE Protein Sample Loading Buffer, 5×	Beyotime Biotechnology	Cat# P0285-15ml
Phosphatase inhibitors and Protein inhibitors mixtures	NCM	Cat# P002
Prestained Protein Ladder	Thermo Fisher Scientific	Cat# 26616
Skim milk powder	Applygen	Cat# P1622
NcmBlot Rapid transfer buffer (20×)	NCM Biotech	Cat# WB4600
SDS-PAGE stacking gel buffer (4×)	CWBIO	Cat# CW0026S
SDS-PAGE separating gel buffer (4×)	CWBIO	Cat# CW0025S
30% PAGE Pre-Solution	Solarbio LIFE SCIENCE	Cat# A1010
4′,6-Diamidino-2-phenylindole (DAPI)	Beyotime Biotechnology	Cat# C1005
Dimethyl sulfoxide (DMSO)	Sigma–Aldrich	Cat# 102623624
Hoechst 33342	Beyotime Biotechnology	Cat# C11028
DEPC-treated water	Solarbio LIFE SCIENCE	Cat# R0601
Triton X-100	Yeasen	Cat# 20107ES76
L-Glutamine (^{13}C5, 99%)	Cambridge isotope	Cat# CLM-1822-H-0.1
L-Aspartic acid (^{13}C,^{15}N)	Sigma–Aldrich	Cat# 607835
1M Hepes (pH7.2–7.4)	Solarbio LIFE SCIENCE	Cat# H1095
Tris-Glycine SDS-PAGE running buffer (Powder)	Servicebio	Cat# G2018-15
TBS-T	NevBio	Cat# NP2111M

1% crystal violet	Solarbio LIFE SCIENCE	Cat# G1062
PBS	Solarbio LIFE SCIENCE	Cat# P1020
Propidium iodide (PI)	Solarbio LIFE SCIENCE	Cat# C0080
TBTA	GLPBIO	Cat# GC45003-50
TCEP	Sigma–Aldrich	Cat# C4706
**Critical commercial kits**		
Cell Counting Kit-8	Beyotime Biotechnology	C0040
BCA Protein Assay Kit	Beyotime Biotechnology	P0011
Animal RNA isolation kit with spin column	Beyotime Biotechnology	R0026
Endofree maxi plasmid kit	Tiangen	DP117
HiFscript gDNA Removal RT MasterMix	CWBIO	CW2020M
MagicSYBR mixture	CWBIO	CW3008M
Universal virus concentration kit	Beyotime Biotechnology	C2901S
Super ECL Detection Reagent	Yeasen	36208ES60
Membrane and Cytosol Protein Extraction kit	Beyotime Biotechnology	P0033
Cell-light EdU Apollo567 In Vitro Kit	RIBOBIO	C10310-1
Lipofectamine 3000 Transfection Kit	Thermo Fisher Scientific	L3000015
RT cDNA Synthesis Kit	CWBIO	CW0741M
**Cell lines**		
Mouse: GL261	BNCC	Cat# BNCC341792
Human: U87-MG	Pricella	Cat# CL-0238
Human: HEK293T	ATCC	Cat# CBP60439
Human: U251-MG	Provided by the Cancer Hospital of the Chinese Academy of Medical Sciences.	
**Viruses and plasmids**		
Virus: sgNC	Genechem	N/A
Virus:sgSCAMP2#2	Genechem	N/A
Virus:sgSCAMP2#3	Genechem	N/A
SCAMP2 pcDNA3.1-his-C	Youbio	N/A
SCAMP2 1–151 pcDNA3.1-his-C	Youbio	N/A
SCAMP2 PCMV6-MYC-C	Youbio	N/A
SLC25A12 pcDNA3.1-3×Flag-C	Youbio	N/A
SLC1A3 pcDNA3.1-3×Flag-C	Youbio	N/A
SLC25A12 pcDNA3.1-mCherry-C2	Youbio	N/A
SLC1A3 pcDNA3.1-mCherry-C2	Youbio	N/A
SCAMP2 pcDNA3.1-EGFP-C2	Youbio	N/A
SCAMP2 pcDH-GFP-PURO-3×Flag	Youbio	N/A
**Others**		
0.45 µm PVDF membrane	Millipore	Cat# IPVH00010
Anti-His magnetic beads	Beyotime Biotechnology	Cat# P2135
Anti-Flag magnetic beads	Selleck	Cat# B26101
Anti-Myc magnetic beads	Selleck	Cat# B26301
Matrigel	Corning	Cat# 356234

Table S3. ^{1}H NMR and ^{13}C NMR spectroscopic data (δ) for compounds AUX-B and AUX-B-p (δ in ppm).

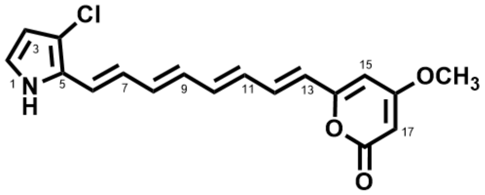
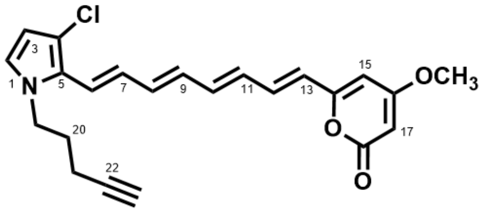

	δ_H (mult, *J* in Hz)		δ_C	
No.	**AUX-B**	**AUX-B-p**	**AUX-B**	**AUX-B-p**
1	11.46 (br, s)	---	---	---
2	6.89 (t, 3,)	6.90 (d,2.8)	120.4	123.6
3	6.12 (t, 3)	6.15 (d, 2.8)	109.2	109.2
4	---	---	111.9	111.0
5	---	---	126.0	124.8
6	6.53 (d, 15)	6.69 (d, 14)	120.8	120.9
7	6.76 (dd, 15, 10)	7.03 (dd, 14,14)	124.7	127.3
8	6.61 (dd, 15, 10)	6.61 (dd, 14,14)	136.9	137.5
9	6.40 (dd, 15, 10)	6.50 (dd, 14,14)	131.5	131.9
10	6.72 (dd, 15, 10)	6.75 (dd, 14,14)	138.9	138.8
11	6.47 (dd, 15, 10)	6.46 (dd, 14,14)	130.5	130.8
12	7.04 (dd, 15, 10)	7.06 (dd, 14,14)	135.2	135.1
13	6.29 (d, 15)	6.31 (d,14)	121.6	121.8
14	---	---	158.4	158.4
15	6.24 (d, 2.0)	6.24 (d, 2.1)	100.8	100.8
16	---	---	170.9	170.8
16-OMe	3.81 (s)	3.81(s)	56.5	56.4
17	5.59 (d, 2.0)	5.59 (d, 2.1)	88.4	88.5
18	---	---	162.6	162.7
19		4.04 (t, 7)		46.2
20		1.78 (m)		29.5
21		2.12 (m)		14.9
22		---		83.3
23		2.91 (s)		72.4

NMR data (δ) were measured at 500 MHz/125 MHz in DMSO-d_6 for **AUX-B** and **AUX-B-p**.
